# Evaluation of the draft guidelines proposed by EMA and FDA for the clinical diagnosis of acute uncomplicated cystitis in women

**DOI:** 10.1007/s00345-019-02761-3

**Published:** 2019-04-19

**Authors:** Jakhongir F. Alidjanov, Kurt G. Naber, Adrian Pilatz, Abdukhamid Radzhabov, Musluhuddin Zamuddinov, András Magyar, Peter Tenke, Florian M. Wagenlehner

**Affiliations:** 1 Department of Urology, Pediatric Urology and Andrology, Justus-Liebig-University, Rudolph-Buchheim Str.7, 35392 Giessen, Germany; 2 Straubing, Germany; 3 Treatment and Diagnostic Center “Olami Tib”, J.Rasulov 29 Street., 734060 Dushanbe, Tajikistan; 4 Sankt-Katharinen Hospital, Seckbacher Landstr. 65E, 60389 Frankfurt, Germany; 5 Department of Urology, Madadi Akbar Clinic, Ayni Street 14, Dushanbe, Tajikistan; 6 Department of Urology, Jahn Ferenc South Pest Teaching Hospital, Köves út 1, Budapest, 1204 Hungary

**Keywords:** Urinary tract infection, Cystitis, Acute Cystitis Symptom Score, ACSS, Guidelines, Diagnosis

## Abstract

**Purpose:**

To reassess the diagnostic values of the “draft” guidelines for the clinical diagnosis of acute uncomplicated cystitis (AC), recently proposed by US Food and Drug Administration (FDA) and European Medicines Agency (EMA).

**Methods:**

The data of 517 female respondents (patients with acute cystitis and controls) derived from the e-USQOLAT database were analyzed and used for the validation of proposed “draft” guidelines of FDA and EMA, compared to the Acute Cystitis Symptom Score (ACSS) questionnaire. The diagnostic values of the proposals concerning signs, symptoms and their severity were assessed and compared.

**Results:**

The six “typical” symptoms of the ACSS were strongly associated with the diagnosis of AC. The number of positive “typical” symptoms differed significantly between patients and controls: median 5 (IQR 4–6) vs 1 (IQR 0–3) respectively. Scored severity of “typical” symptoms also differed significantly between groups of patients and controls: median (IQR) 10 (7–13) vs 1 (0–4), respectively. The best balance between sensitivity and specificity is shown by the ACSS cut-off value of 6 scores and more of the “Typical” domain, followed by an approach proposed by FDA and EMA, justifying ACSS to be used as a diagnostic criterion for the clinical diagnosis of AC.

**Conclusions:**

Not only the presence but also the severity of the symptoms is important for an accurate diagnosis of AC. The ACSS, even without urinalysis is at least as favourable as the draft diagnostic proposals by FDA and EMA. The ACSS can be recommended for epidemiological and interventional studies, and allows women to establish self-diagnosis of AC, making the ACSS also cost-effective for healthcare.

**Electronic supplementary material:**

The online version of this article (10.1007/s00345-019-02761-3) contains supplementary material, which is available to authorized users.

## Introduction

Despite numerous publications, there is still no generally accepted strategy regarding the clinical diagnosis of acute uncomplicated cystitis (AC). The updated guidelines of the Infectious Diseases Society of America (IDSA) and the European Society for Microbiology and Infectious Diseases (ESCMID) mainly consist of recommendations about the treatment of AC and not the diagnosis [1]. These guidelines were limited to the treatment of AC and pyelonephritis in premenopausal, non-pregnant women with no known urological abnormalities or comorbidities. In addition, the authors noted that postmenopausal women or those who have well-controlled diabetes mellitus in the absence of urological sequelae may be considered as having uncomplicated UTIs (uUTIs) by some experts, but a discussion of specific management of these groups was outside the scope of the guidelines.

In the last update of these guidelines of the European Association of Urology (EAU) from 2019, AC is defined as acute, sporadic or recurrent cystitis limited to non-pregnant women with no known relevant anatomical and functional abnormalities within the urinary tract or comorbidities [2]. According to the EAU guidelines, the diagnosis of AC can be made with a high probability based on a focused history of lower urinary tract symptoms (dysuria, frequency and urgency) and the absence of vaginal discharge or irritation.

The definition of UTIs in a broader sense is presented in the updated German National Clinical Practice S3 Guideline [3]: UTIs may be classified as uncomplicated in the absence of relevant functional or anatomical abnormalities in the urinary tract, with no relevant renal functional impairment and any relevant concomitant disease that could aggravate the UTIs or condition, which could increase the risk of development of serious complications. Simple cystitis in this regard, may represent no additional health problem for the woman with stable diabetes mellitus, whereas any kind of pyelonephritis, whether earlier defined as uncomplicated or complicated, could interfere with her metabolic balance and could lead to severe complications. It becomes obvious today that a simple general classification of UTIs into uncomplicated and complicated UTIs is far too rough. Therefore, a more differentiated stratification of UTIs with the deeper consideration of risk factors was proposed earlier [4].

Recently, the US Food and Drug Administration (FDA) and European Medicines Agency (EMA) have proposed “draft” guidelines for the clinical diagnosis of patients with AC for further discussion:

- Adult and, if appropriate, adolescent females with evidence of pyuria (WBC ≥ 10/µL) and at least two of the following signs or symptoms of dysuria, urinary frequency, urinary urgency, and suprapubic pain (FDA) [5];
- Female patients with documented pyuria (WBC ≥ 10/µL) and having a minimum number of symptoms such as frequency, urgency and dysuria (EMA) [6].

We aimed to reassess the diagnostic values of these proposed draft guidelines using the Acute Cystitis Symptom Score (ACSS) which was validated in several languages [7–10].

## Material and methods

### Study design

The current study is designed as a non-interventional, case–control study.

### Data acquisition

The e-USQOLAT database, containing the relevant clinical and laboratory data obtained from female respondents (patients with AC and controls without AC) during clinical validation of the ACSS in several countries was selected as a primary source for data mining [11]. All relevant data were acquired from the database at the access date of January 1, 2019.

The “diagnostic Part A” of the ACSS questionnaire, used for diagnostic purposes contains four domains [7]. Since all information essential for our purpose, concerning symptomatology (four symptoms mentioned above, plus two symptoms: “incomplete bladder emptying” and “visible blood in urine”) constitutes the “typical” domain of the ACSS, we decided to limit our analysis of the symptoms and their severity to this domain. Analyses of other items and domains of the ACSS are discussed elsewhere [12].

Further information about the questionnaire itself in different languages can be found on the ACSS website (https://www.acss.world).

### Data processing

Only cases with sufficient information concerning questionnaire data and urinalysis were selected for further statistical analysis.

The diagnosis concerning the presence or absence of AC, made by the treating physician based on the history and the results of the laboratory findings in accordance with national and/or international standards and guidelines [1–3] was taken as reference. Confirmed diagnosis of AC was considered a positive diagnostic outcome (patients) and the absence of AC was taken as a negative diagnostic outcome (controls), respectively.

The presence of symptoms (positive, negative), symptoms’ severity (mild, moderate, severe), and the proposed diagnostic approaches (EMA, FDA, ACSS) were considered for calculation of their diagnostic values.

Presence of pyuria was considered a confounder. Since two different types of urinalyses were performed in different countries (dipsticks with esterase test or microscopy according to Nechiporenko [13]), results of these two methods were unified and labelled, respectively, as “negative”, “trace”, “small”, “moderate” and “large”, depending on the number of white blood cells.

Data processing included a procedure of dichotomization of variables for the assessment of diagnostic values. Generally, relative variables were labelled as “0” for “negative”/”not match”, and “1” for “positive”/”match”.

### Statistical analysis

Contingency tables were used for the statistical analysis of the bivariate (dichotomized) variables. The diagnostic values of the different proposals regarding the relations of exposure, confounder and the diagnostic outcome were assessed. Values such as sensitivity, specificity, positive and negative likelihood ratios (+ LR and − LR, respectively), Youden’s J-index, diagnostic odds ratio (DOR), positive and negative predictive values (PPV and NPV, respectively) were calculated. ROC curve analysis was used for the assessment of area under the curve (AUC). The strength of associations between exposure and a positive diagnostic outcome was measured using Pearson’s product-moment correlation coefficient.

Tests of the comparative analyses were performed in dependence of normality and homoscedasticity of distributions which in turn were assessed using dot charts and *Q*–*Q* plots.

For the comparison of independent, homoscedastic and normally distributed variables, Student’s two-sided *t* test was used. For normally distributed heteroscedastic independent variables, Welch’s two-sided modified *t* test was used. Non-parametric tests were used when parametric tests were considered inappropriate. A *p* value of less than 0.05 was considered statistically significant.

R v.3.5.2 with in-built and additional (third-party) packages was used for the statistical analysis and graphical representation of the results [14–16].

## Results

**Fig. 1:**
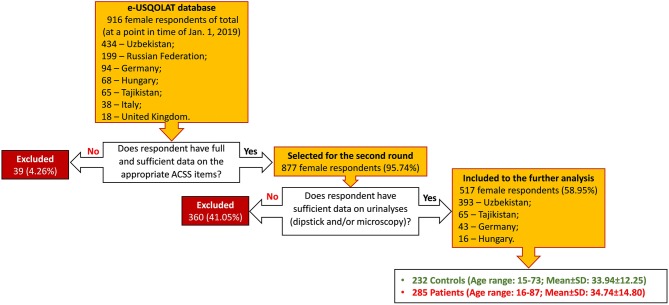
Flowchart of the selection of the study population

On the access date, the e-USQOLAT database contained information about 911 female respondents from seven different countries (Fig. 1). Respondents are allocated to the groups of patients (with AC) and controls (without AC) according to the final diagnosis of the treating physician.

A total number of 517 respondents from four countries matched all the inclusion criteria and could be selected for further data processing and analysis (Fig. 1). Missing results of urinalysis accounted for the majority of mismatches in the inclusion criteria (360; 39.52% of total). Only 39 of excluded respondents had no sufficient questionnaire data (4.28% of total).

The age of the population included in the study ranged from 15 to 87 years with the following averages: median (interquartile range—IQR) − 30.50 (24.00; 40.00), mean ± SD − 34.38 ± 13.71. The group of controls consisted of 232 (44.87%) respondents with a median age (IQR) − 31.00 (25.00; 40.00), a mean age ± SD - 33.94 ± 12.25, ranging from 15 to 73 years. Two hundred eighty-five (55.13%) respondents in the group of patients had a median age (IQR) of 30.00 (24.00; 41.00), a mean age ± SD − 34.74 ± 14.80, ranging from 18 to 87 years old. The process of selection of the study population and essential demographic data are presented in Fig. 1 and Table 1.

**Table 1:** Demographics of the study population (patients with AC and controls without AC)

	Total *N* = 517		Controls *N* = 232		Patients *N* = 285	
	*N*	Prevalence among the study population	*N*	Prevalence within the group	*N*	Prevalence within the group
Parameter						
Age						
Young girls (15–21 years old)	73	14.15	31	13.36	42	14.74
First mature age (22–35 years old)	254	49.22	117	50.43	137	48.07
Second mature age (36–55 years old)	134	25.97	63	27.16	71	24.91
Advanced age (56–74 years old)	50	9.69	20	8.62	30	10.53
Old age ( ≥ 74 years old)	5	0.97	0	0.00	5	1.75
Language versions of the ACSS filled						
Uzbek (cyr)	294	56.87	140	60.34	154	54.04
Russian	87	16.83	44	18.97	43	15.09
Tajik	58	11.22	21	9.05	37	12.98
German	43	8.32	19	8.19	24	8.42
Uzbek (lat)	19	3.68	4	1.72	15	5.26
Hungarian	16	3.09	4	1.72	12	4.21
Additional conditions at the time of visit						
Pregnancy	58	11.22	27	11.64	31	10.88
Symptoms of the menopause	43	8.32	21	9.05	22	7.72
Menstruation ("monthlies")	46	8.90	19	8.19	27	9.47
Signs of premenstrual syndrome (PMS)	43	8.32	18	7.76	25	8.77
Known sugar diabetes	4	0.77	2	0.86	2	0.70
Pyuria	306	59.19	64	27.59	242	84.91

*AC* acute uncomplicated cystitis

Linear model fit analysis for “diagnostically significant grades” of pyuria revealed values of ≥ 25 WBC/µL for dipstick analysis and > 8000 WBC/mL for urine microscopy according to Nechiporenko [13] to have a statistically significant positive relationship with the diagnosis of AC: sensitivity − 0.85 [95% CI = 0.80; 0.89], specificity − 0.72 [0.66; 0.78], PPV − 0.79 [0.74;0.84], NPV − 0.80 [0.74; 0.85], crude DOR − 14.77 [9.57; 22.80], Youden index − 0.57 [0.46; 0.67].

**Fig. 2:**
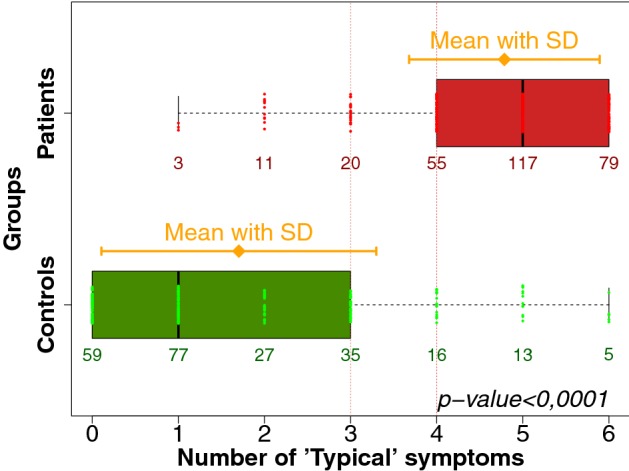
Boxplots (IQR, range, mean ± SD) of the number of the ACSS typical symptoms in respondents (Patients with AC, Controls without AC)

The median number of positive symptoms for controls was 1 with IQR of 0–3 and differed significantly non-significant (*p* < 0.001) from that for patients, which was 5 with IQR of 4–6 (Fig. 2).

According to the ACSS data, the most common symptom among the entire study population was urinary frequency (72.92%). It included 47.84% of controls and 93.33% of patients. Whereas the majority of controls experienced “mild” urinary frequency (81/111 = 72.97%), “moderate” or “severe” values of the symptom were more “specific” for the group of patients (189/266 = 71.05%) (Table 2).

**Table 2:** ACSS parameters of the study population (patients with AC and controls without AC)s

	Total *N* = 517		Controls *N* = 232		Patients *N* = 285		
	*N*	Prevalence among study population	*N*	Prevalence within the group	*N*	Prevalence within the group	
Urinary frequency	377	72.92	111	47.84	266	93.33	
Mild	158	30.56	81	34.91	77	27.02	
Moderate	118	22.82	24	10.34	94	32.98	
Severe	101	19.54	6	2.59	95	33.33	
Urinary urgency	313	60.54	63	27.16	250	87.72	
Mild	88	17.02	40	17.24	48	16.84	
Moderate	114	22.05	14	6.03	100	35.09	
Severe	111	21.47	9	3.88	102	35.79	
Dysuria	306	59.19	48	20.69	258	90.53	
Mild	83	16.05	29	12.50	54	18.95	
Moderate	102	19.73	10	4.31	92	32.28	
Severe	121	23.40	9	3.88	112	39.30	
Suprapubic pain	319	61.70	82	35.34	237	83.16	
Mild	121	23.40	45	19.40	76	26.67	
Moderate	124	23.98	27	11.64	97	34.04	
Severe	74	14.31	10	4.31	64	22.46	
Sense of incomplete bladder emptying	319	61.70	69	29.74	250	87.72	
Mild	114	22.05	43	18.53	71	24.91	
Moderate	121	23.40	20	8.62	101	35.44	
Severe	84	16.25	6	2.59	78	27.37	
Visible blood in the urine	125	24.18	22	9.48	103	36.14	
Mild	64	12.38	11	4.74	53	18.60	
Moderate	37	7.16	7	3.02	30	10.53	
Severe	24	4.64	4	1.72	20	7.02	

*AC* acute uncomplicated cystitis

**Fig. 3:**
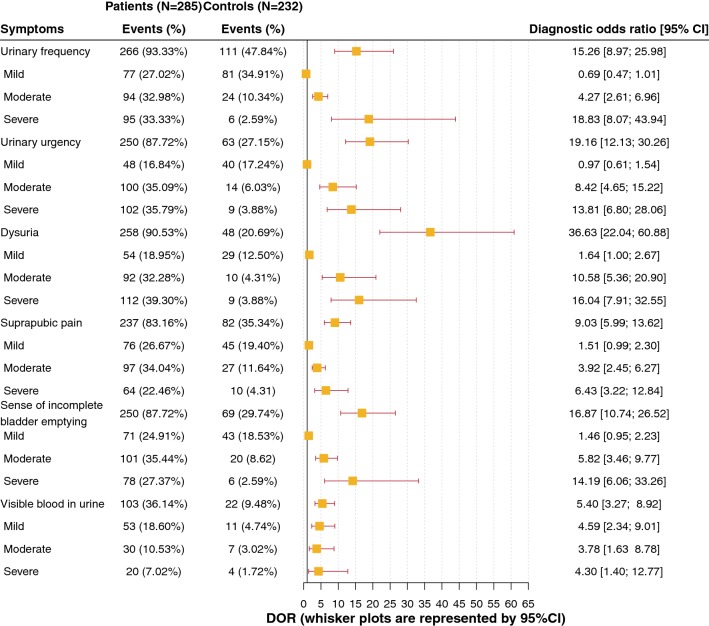
Prevalence and diagnostic odds ratio (average, 95% CI) of the six ACSS typical symptoms in the study population (patients with AC and controls without AC)

**Fig. 4:**
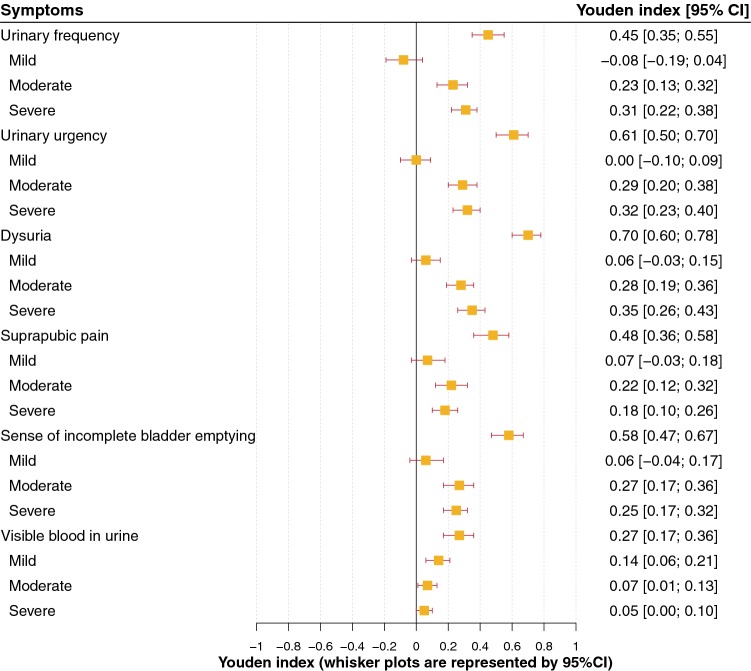
Youden’s index of the six ACSS typical symptoms according to presence and severity in the study population (patients with AC and controls without AC)

Figures 3 and 4, respectively, represent the prevalence, DOR and Youden’s index of the six “typical” symptoms and their severity, used in the ACSS questionnaire. All six symptoms had a significant positive association with a positive outcome (PO), i.e. diagnosis of AC. It also was verified that not only the presence of the symptoms but also their severity is important for the diagnosis (Fig. 4). More detailed results of the analysis of different diagnostic values of these symptoms and their severity are given in Supplementary Tables 1, 2, 3.

**Fig. 5:**
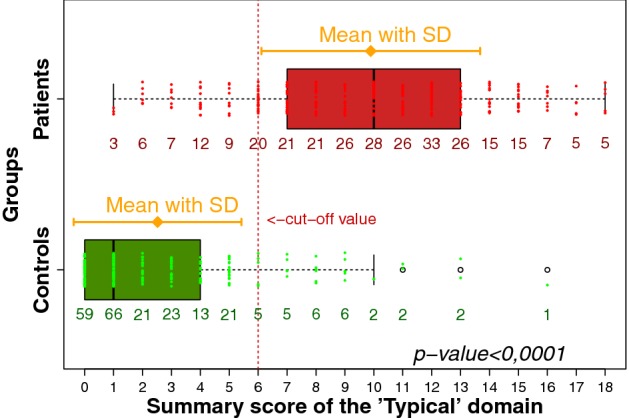
Boxplots (IQR, range, mean ± SD) of the summary score of the six ACSS typical symptoms in respondents (patients with AC, controls without AC)

Scoring the symptoms into 0 (no symptom), 1 (mild), 2 (moderate), and 3 (severe) revealed for controls a median symptom score of 1 with IQR of 0–4 which significantly differed from that for patients: 10 with IQR of 7–13 (*p* < 0.001) (Fig. 5).

**Fig. 6:**
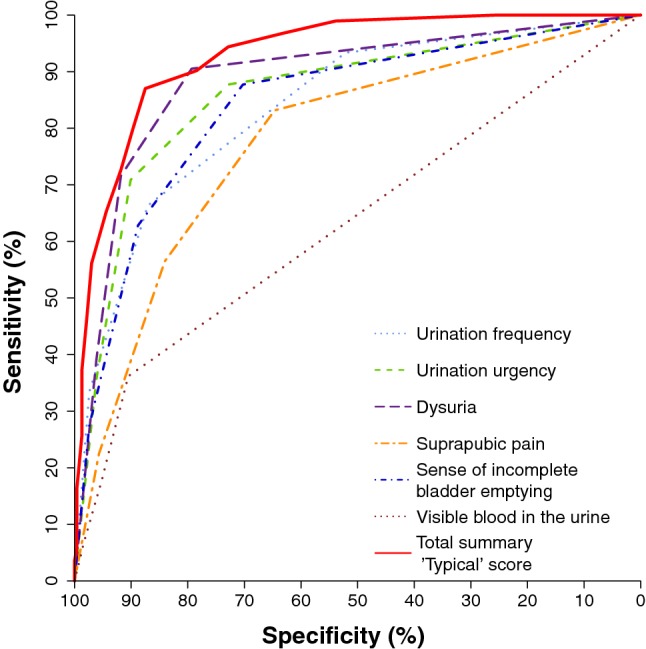
Receiver operating characteristic (ROC) curves for the six individual typical symptoms and the summary score of the six symptoms proposed by ACSS

ROC curve analysis revealed the largest area under the curve (AUC) for the summary score of the “typical” domain of the ACSS (AUC [95% CI] = 0.93 [0.91; 0.95]), in descending order followed by dysuria (0.85 [0.82; 0.88]), urination urgency (0.85 [0.82; 0.88]), sense of incomplete bladder emptying (0.79 [0.75; 0.83]), suprapubic pain (0.74 [0.70; 0.78]), and visible blood in urine (0.63 [0.60; 0.67]) (Fig. 6).

Sensitivity and specificity (average [95% CI]) for the different proposed approaches of diagnosing AC are the following:

- 0.84 [0.79; 0.88] and 0.83 [0.77; 0.87] for the draft approach by EMA^1^;
- 0.83 [0.78; 0.87] and 0.88 [0.84; 0.92] for the draft approach by FDA^2^; and
- 0.87 [0.83; 0.91] and 0.88 [0.83; 0.91] for the cut-off value of the ACSS^3^, respectively.

The differences in diagnostic values between these three diagnostic approaches are, however, statistically not significant (*p* > 0.05) (Supplementary Tables 2 and 3).

If the cut-off value of the ACSS is combined with positive pyuria, then the specificity and sensitivity change to 0.96 [0.93; 0.98] and 0.73 [0.67; 0.78], respectively.

Pyuria by itself had a reasonable sensitivity (0.85 [0.80; 0.89]) and specificity (0.72 [0.66; 0.78]) (Suppl. Table 2).

The ROC curve analysis of the proposed diagnostic approaches demonstrated the best balance between sensitivity and specificity in the following descending order: ACSS cut-off value of ≥ 6 of “typical” domain (AUC [95% CI] of 0.87 [0.84; 0.90]), draft proposal by FDA (0.85 [0.82; 0.88]), and the draft proposal by EMA (0.83 [0.80; 0.87]). However, the differences in AUC between the three mentioned approaches were statistically non-significant (*p* > 0.05).

Diagnostic values of different numbers and scores of symptoms with or without considering pyuria are presented in Supplementary Tables 2 and 3. Graphical representation of the different diagnostic proposals by FDA, EMA, and ACSS is given as Supplementary Figs. 1, 2, 3, 4, 5, 6, 7, 8, 9.

## Discussion

Urinary tract infections (UTIs) are among the most widespread infectious diseases in general practice [17], with 80% of cases classified as uUTIs. Although current guidelines recommend antibiotics as the first choice of treatment for the acute phase [2, 18], several prospective randomized, placebo-controlled studies comparing antibiotic and non-antimicrobial symptomatic therapeutic modalities have been performed [19–22]. Results of these studies were compelling enough for the updated German Clinical Guidelines [18] to encourage the use of the non-AB symptomatic treatment in selected cases of acute lower uUTIs with mild-to-moderate symptoms.

Since AC can be considered a benign infection without general risk of aggravation of UTI or serious complications, mainly the clinical diagnosis with or without point of care urinalysis (such as pyuria) and longer term follow-up with patient-reported clinical outcome (e.g. for at least 4 weeks after end of treatment) should become the main inclusion and outcome criteria of future studies. This would also better correspond to the general recommendations and everyday practice, making urine culture unnecessary, with the exception of specific situations, such as (a) suspected pyelonephritis, (b) symptoms not resolving within about 1 week or recurring within 4 weeks after the completion of treatment; (c) atypical symptoms; (d) pregnancy [2].

Urine culture before and probably after treatment will remain important for epidemiological studies, and for studies including at least one antimicrobial therapy arm. However, the use of any defined significant bacteriuria as post hoc inclusion criterion is at least questionable. Nowadays, it is known that even under normal physiological condition, urine is not sterile [23]. The term “significant bacteriuria” was used in the past to differentiate between infection and contamination of a urine sample collected for analysis. When bladder urine from patients with unquestioned acute pyelonephritis was examined quantitatively, none contained less than 10^5^ colony forming units (CFU) of uropathogen per mL [24]. Bacteriuria of ≥ 10^5^ CFU/mL in adults was originally defined significant only for the diagnosis of pyelonephritis. In 1982, Stamm et al. [25] documented that the levels of ≥ 10^5^ CFU/mL of a pathogen in urine have a very high specificity (99%) but a very low sensitivity (51%) for the diagnosis of AC. Bacteriuria of ≥ 10^2^ CFU/mL was suggested by the authors as the best diagnostic criterion (sensitivity, 95%; specificity, 85%). In 2013, Hooton et al. [26] confirmed that *E. coli* identified as low as 10^1^–10^2^ CFU/mL was sensitive and specific for the diagnosis of AC in symptomatic women. But still, about 20% of these symptomatic female patients were culture “negative” even when being tested for such low counts. Quantitative PCR (qPCR) for *E. coli* and *S. saprophyticus* finally demonstrated that almost all women with symptoms suggestive for UTIs and a “negative” culture still have an infection with *E. coli* [27]. Therefore, according to the German National S3 Guideline, the detection of *E. coli* in symptomatic women is predictive for a bacterial UTI, irrespective of the number of pathogens. In contrast, the presence of Enterococci and group B *Streptococci* in urine is not predictive for UTIs [3].

Hence, the use of a general definition for significant bacteriuria of ≥ 10^5^ CFU/mL as an inclusion criterion, may falsely exclude about half of the patients with a probable diagnosis of AC presented with the same symptoms. Therapeutic consequences drawn from such studies may have to be then restricted for this subgroup of patients. Therefore, we recommend considering all patients included with the same clinical criteria into a study as the main target population. Patients with bacteriuria of ≥ 10^2^ or ≥ 10^3^ CFU/mL, in turn, should then only be considered as microbiologically evaluable patients. The same principles should be applied for outcome criteria, based on patient-reported outcome using a validated questionnaire at least up to 4 weeks after the end of therapy. Consideration of the elimination of bacteriuria as the main study aim is scientifically questionable, due to the findings that asymptomatic bacteriuria may probably be protective against recurrent UTI [28, 29]. It should, however, be registered as additional results of the study.

The analysis of 517 female respondents (patients and controls) has revealed that the diagnostic value of the ACSS cut-off value without urinalysis is at least as favourable as the draft proposals by FDA or by EMA. The most important advantage of the ACSS is that it could be used also in epidemiological studies or for self-diagnosis of the patient without the need for additional laboratory tests, such as urinalysis. For clinical interventional studies, however, the same threshold could be used as an inclusion criterion together with the evidence of pyuria and thus dramatically increasing the specificity.

Although it has been demonstrated that the scoring of the five first typical symptoms in the ACSS questionnaire are not much inferior to the six symptoms, including visible blood, we recommend to include further all six items in the typical domain, because visible hematuria in connection with typical urinary symptoms may be pathognomonic for acute hemorrhagic cystitis. It can also be an important differential sign. If visible hematuria persists after treatment, it needs a further careful investigation of the patient to exclude any other urological disease, such as bladder cancer. The Swiss guidelines have also included a recent onset of hematuria as one of the typical symptoms of AC besides frequency, urgency and dysuria with pyuria and bacteriuria of ≥ 10^2^ CFU/ml [30].

The shortcoming of the study is mainly related to the design as a non-interventional, case–control study.

There are different laboratory methods defining pyuria in different countries. The dichotomized approach allocating pyuria into “significant” and “non-significant” allowed to bring the values together, thus reducing possible biases. The difference between pyuria (WBC ≥ 25/µL) tested in the current analysis and pyuria (WBC ≥ 10/µL) proposed by FDA and EMA remains open. The ratio of patients and controls in our study is 1:0.81, which is close to the optimal ratio of 1:1.

Because of the non-interventional approach, the study protocol could only be suggestive for the participating physicians, who were asked to follow the national and international guidelines for the diagnosis and treatment of women with AC. Therefore, variations of the management could only be minimized but not completely avoided.

## Conclusions

The diagnostic values of the “draft” guidelines proposed by FDA and EMA were compared with the validated ACSS questionnaire. Not only the presence but also the severity of the symptoms (scoring) are important for an accurate diagnosis of AC. It could be shown that the diagnostic value of the ACSS, even without additional urinalysis, is at least as favorable for the clinical diagnosis of AC as the draft clinical proposals by FDA and EMA. Therefore, the ACSS can be recommended for epidemiological and interventional studies, and allows women for self-diagnosis of AC, which makes the ACSS also cost-effective for healthcare.

## Electronic supplementary material

Below is the link to the electronic supplementary material.

Supplementary file1 (DOCX 407 kb)

## Footnotes

1 A minimum number of symptoms such as frequency, urgency and dysuria AND documented pyuria.

2 At least two of such symptoms as dysuria, urinary frequency, urinary urgency, and suprapubic pain AND evidence of pyuria.

3 Summary score of “Typical” domain ≥ 6.
