# Supplementary material for: Evaluation of the draft guidelines proposed by EMA and FDA for the clinical diagnosis of acute uncomplicated cystitis in women
Source: World J Urol. 2019 Apr 19;38(1):63–72. doi: 10.1007/s00345-019-02761-3 (PMC6954149; doi:10.1007/s00345-019-02761-3)
Supplement: Supplementary file 1 — Supplementary file1 (DOCX 407 kb) [file 345_2019_2761_MOESM1_ESM.docx]

Supplementary Figure 1. Receiver operating characteristic (ROC) curves using the minimal number of typical symptoms proposed by EMA, by FDA, and by ACSS, respectively, with and without considering pyuria.

**
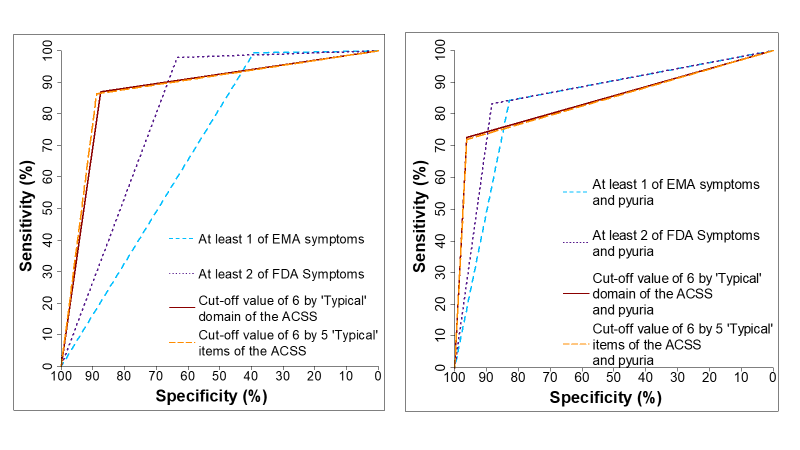
**

Supplementary Figure 2. Receiver operating characteristic (ROC) curves using a summary score of >6 for the 3 symptoms proposed by EMA, for the 4 symptoms proposed by FDA, and for the 5 and 6 symptoms proposed by ACSS, respectively, with and without considering pyuria.

**
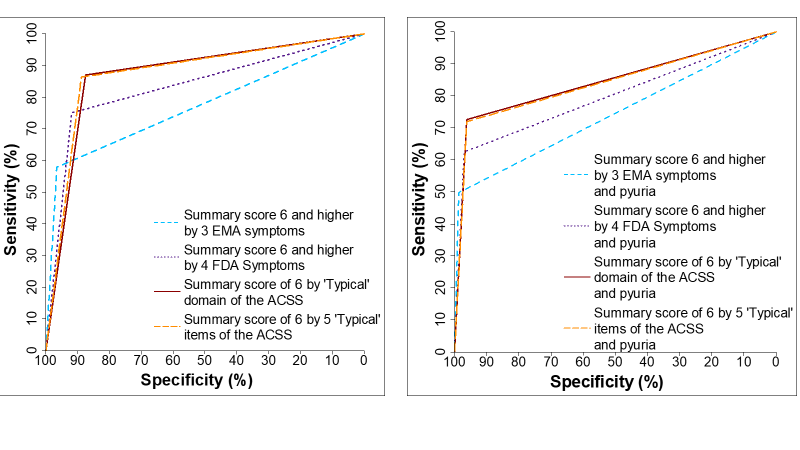
**

Supplementary Figure 3. Youden’s index (mean, 95% CI) of the different draft diagnostic proposals by EMA (at least one of three symptoms), FDA (at least two of four symptoms), and ACSS (cut-off) in the study population (patients with AC and controls without AC) with and without pyuria.

**
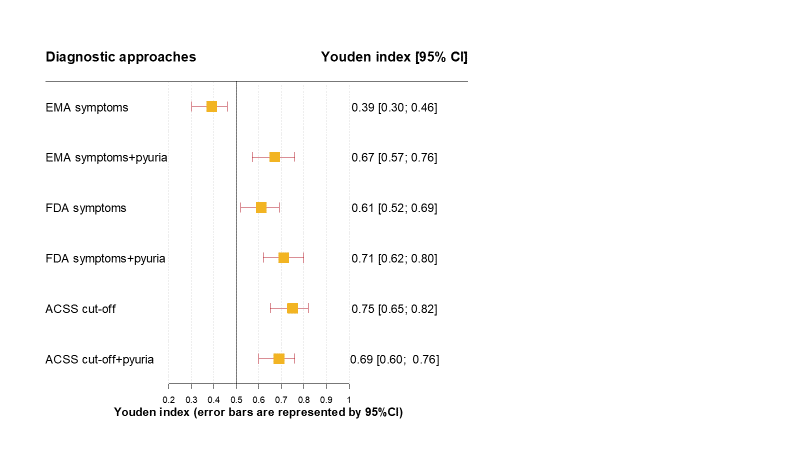
**

**
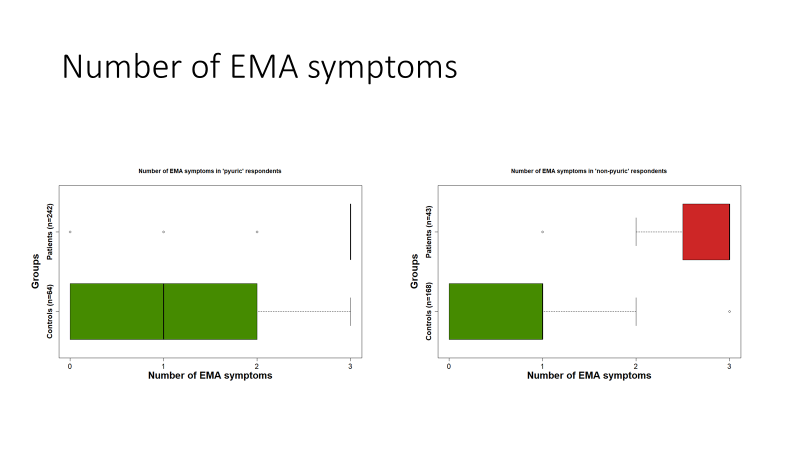
**Supplementary Figure 4. Boxplots (IQR, range) of the number of the EMA typical symptoms in respondents (patients with AC, controls without AC) with and without pyuria

**
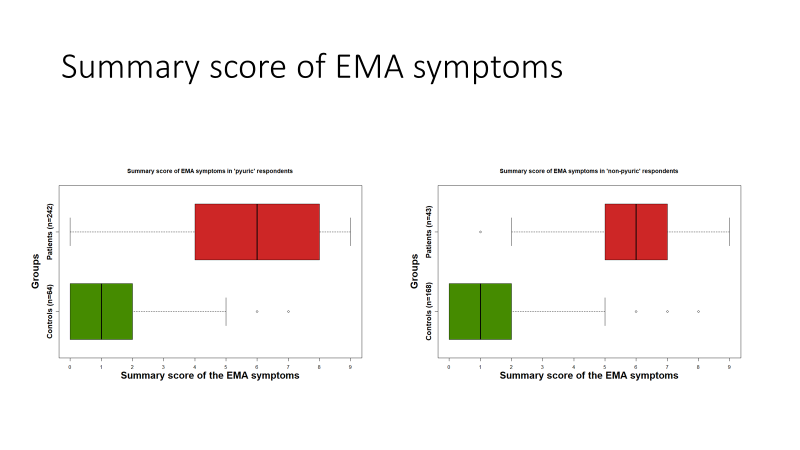
**Supplementary Figure 5. Boxplots (IQR, range) of the summary score of the three EMA typical symptoms in respondents (patients with AC, controls without AC) with and without pyuria

Supplementary Figure 6. Boxplots (IQR, range) of the number of the FDA typical symptoms in respondents (patients with AC, controls without AC) with and without pyuria

**
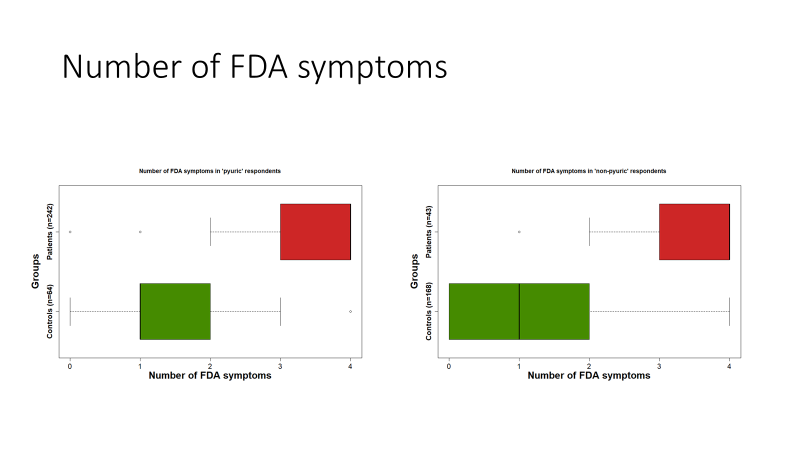
**

Supplementary Figure 7. Boxplots (IQR, range) of the summary score of the four FDA typical symptoms in respondents (patients with AC, controls without AC) with and without pyuria

**
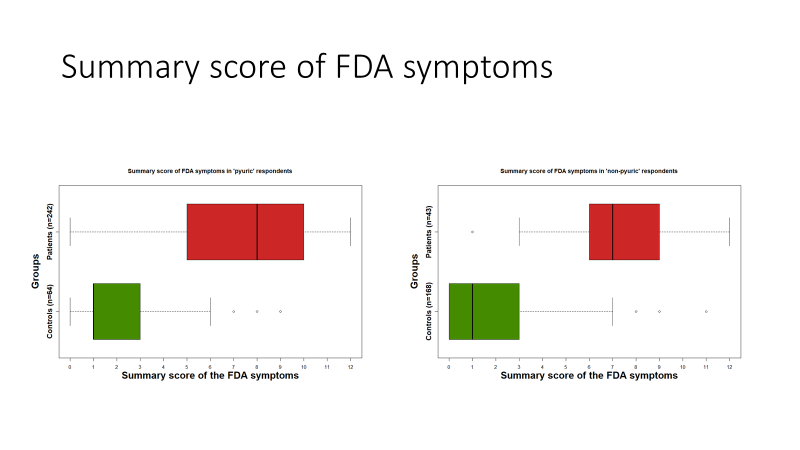
**

**
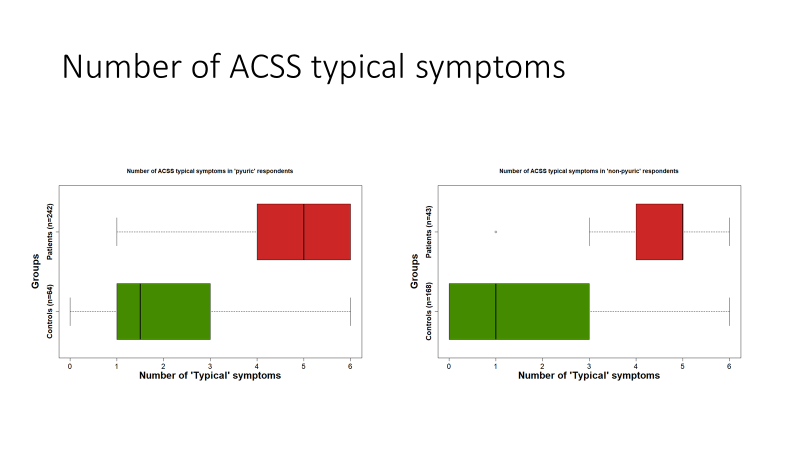
**Supplementary Figure 8. Boxplots (IQR, range) of the number of ACSS typical symptoms in respondents (patients with AC, controls without AC) with and without pyuria

Supplementary Figure 9. Boxplots (IQR, range) of a summary score of the six ACSS typical symptoms in respondents (patients with AC, controls without AC) with and without pyuria

**
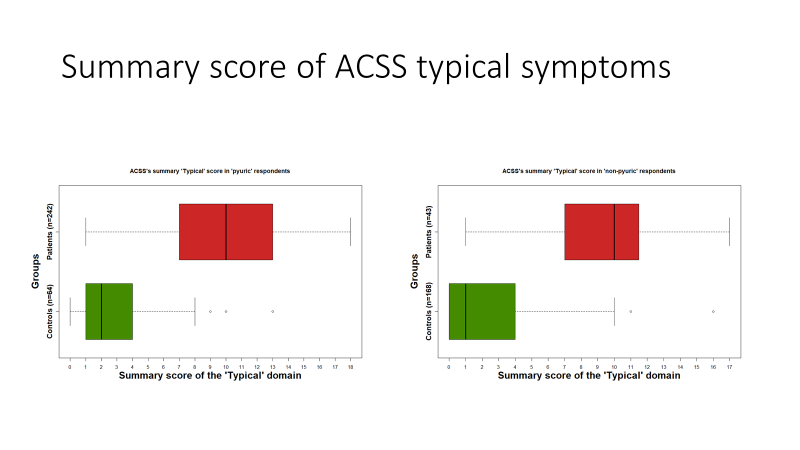
**

Supplementary Table 1. Sensitivity, specificity, positive and negative predictive values (+LR, -LR), and correlation with positive outcome (PO for diagnosis of cystitis of the six ACSS typical symptoms according to the presence (total) and severity. Average value [95% confidence interval]

| **Symptom** | **Sensitivity** | **Specificity** | **PPV** | **NPV** | **+LR** | **-LR** | **DOR** | **Youden's index** | **AUC** | **Correlation with PO** |
| --- | --- | --- | --- | --- | --- | --- | --- | --- | --- | --- |
| **1. Urinary frequency** | 0.93 [0.90; 0.96] | 0.52 [0.46; 0.59] | 0.71 [0.66; 0.75] | 0.86 [0.80; 0.92] | 1.95 [1.70; 2.24] | 0.13 [0.08; 0.20] | 15.26 [8.96; 25.98] | 0.45 [0.35; 0.55] | 0.73 [0.69; 0.76] | 0.51 [0.44; 0.57] |
| Mild | 0.27 [0.22; 0.33] | 0.65 [0.59; 0.71] | 0.49 [0.41; 0.57] | 0.42 [0.37; 0.47] | 0.77 [0.60; 1.00] | 1.12 [1.00; 1.26] | 0.69 [0.47; 1.01] | -0.08 [-0.19; 0.04] | 0.46 [0.42; 0.50] | -0.08 [-0.17; 0.00] |
| Moderate | 0.33 [0.28; 0.39] | 0.90 [0.85; 0.93] | 0.80 [0.71; 0.87] | 0.52 [0.47; 0.57] | 3.19 [2.11; 4.82] | 0.75 [0.68; 0.82] | 4.27 [2.61; 6.96] | 0.23 [0.13; 0.32] | 0.61 [0.58; 0.65] | 0.27 [0.19; 0.35] |
| Severe | 0.33 [0.28; 0.39] | 0.97 [0.94; 0.99] | 0.94 [0.88; 0.98] | 0.54 [0.49; 0.59] | 12.89 [5.75; 28.88] | 0.68 [0.63; 0.74] | 18.83 [8.07; 43.94] | 0.31 [0.22; 0.38] | 0.65 [0.62; 0.68] | 0.39 [0.31; 0.46] |
| **2. Urinary urgency** | 0.88 [0.83; 0.91] | 0.73 [0.67; 0.78] | 0.80 [0.75; 0.84] | 0.83 [0.77; 0.88] | 3.23 [2.60; 4.01] | 0.17 [0.12; 0.23] | 19.16 [12.13; 30.26] | 0.61 [0.50; 0.70] | 0.80 [0.77; 0.84] | 0.62 [0.56; 0.67] |
| Mild | 0.17 [0.13; 0.22] | 0.83 [0.77; 0.87] | 0.55 [0.44; 0.65] | 0.45 [0.40; 0.50] | 0.98 [0.67; 1.43] | 1.00 [0.93; 1.09] | 0.97 [0.61; 1.54] | 0.00 [-0.10; 0.09] | 0.50 [0.47; 0.53] | 0.00 [-0.09; 0.08] |
| Moderate | 0.35 [0.30; 0.41] | 0.94 [0.90; 0.97] | 0.88 [0.80; 0.93] | 0.54 [0.49; 0.59] | 5.81 [3.42; 9.90] | 0.69 [0.63; 0.76] | 8.42 [4.65; 15.22] | 0.29 [0.20; 0.38] | 0.65 [0.61; 0.68] | 0.35 [0.27; 042] |
| Severe | 0.36 [0.30; 0.42] | 0.96 [0.93; 0.98] | 0.92 [0.85; 0.96] | 0.55 [0.50; 0.60] | 9.23 [4.77; 17.83] | 0.67 [0.61; 0.73] | 13.81 [6.80; 28.06] | 0.32 [0.23; 0.40] | 0.66 [0.63; 0.69] | 0.39 [0.31; 0.46] |
| **3. Dysuria** | 0.91 [0.87; 0.94] | 0.79 [0.74; 0.84] | 0.84 [0.80; 0.88] | 0.87 [0.82; 0.91] | 4.38 [3.39; 5.64] | 0.12 [0.08; 0.17] | 36.63 [22.04; 60.88] | 0.70 [0.60; 0.78] | 0.85 [0.82; 0.88] | 0.71 [0.66; 0.75] |
| Mild | 0.19 [0.15; 0.24] | 0.88 [0.83; 0.91] | 0.65 [0.54; 0.75] | 0.47 [0.42; 0.52] | 1.52 [1.00; 2.30] | 0.93 [0.86; 1.00] | 1.64 [1.00; 2.67] | 0.06 [-0.03; 0.15] | 0.53 [0.50; 0.56] | 0.09 [0.00; 0.17] |
| Moderate | 0.32 [0.27; 0.38] | 0.96 [0.92; 0.98] | 0.90 [0.83; 0.95] | 0.53 [0.49; 0.58] | 7.49 [3.99; 14.05] | 0.71 [0.65; 0.77] | 10.58 [5.36; 20.90] | 0.28 [0.19; 0.36] | 0.64 [0.61; 0.67] | 0.35 [0.27; 0.42] |
| Severe | 0.39 [0.34; 0.45] | 0.96 [0.93; 0.98] | 0.93 [0.86; 0.97] | 0.56 [0.51; 0.61] | 10.13 [5.25; 19.53] | 0.63 [0.57; 0.70] | 16.04 [7.91; 32.55] | 0.35 [0.26; 0.43] | 0.68 [0.65; 0.71] | 0.42 [0.34; 0.48] |
| **4. Suprapubic pain** | 0.83 [0.78; 0.87] | 0.65 [0.58; 0.71] | 0.74 [0.69; 0.79] | 0.76 [0.69; 0.82] | 2.35 [1.96; 2.82] | 0.26 [0.20; 0.34] | 9.03 [5.99; 13.62] | 0.48 [0.36; 0.58] | 0.74 [0.70; 0.78] | 0.49 [0.42; 0.55] |
| Mild | 0.27 [0.22; 0.32] | 0.81 [0.75; 0.85] | 0.63 [0.54; 0.71] | 0.47 [0.42; 0.52] | 1.37 [0.99; 1.90] | 0.91 [0.83; 1.00] | 1.51 [0.99; 2.30] | 0.07 [-0.03; 0.18] | 0.54 [0.50; 0.57] | 0.08 [0.00; 0.17] |
| Moderate | 0.34 [0.29; 0.40] | 0.88 [0.84; 0.92] | 0.78 [0.70; 0.85] | 0.52 [0.47; 0.57] | 2.92 [1.98; 4.32] | 0.75 [0.68; 0.82] | 3.92 [2.45; 6.27] | 0.22 [0.12; 0.32] | 0.61 [0.58; 0.65] | 0.26 [0.18; 0.34] |
| Severe | 0.22 [0.18; 0.28] | 0.96 [0.92; 0.98] | 0.86 [0.77; 0.93] | 0.50 [0.45; 0.55] | 5.21 [2.74; 9.92] | 0.81 [0.76; 0.87] | 6.43 [3.22; 12.84] | 0.18 [0.10; 0.26] | 0.59 [0.56; 0.62] | 0.26 [0.17; 0.34] |
| **5. Sense of incomplete bladder emptying** | 0.88 [0.83; 0.91] | 0.70 [0.64; 0.76] | 0.78 [0.73; 0.83] | 0.82 [0.76; 0.87] | 2.95 [2.41; 3.61] | 0.17 [0.13; 0.24] | 16.87 [10.74; 26.52] | 0.58 [0.47; 0.67] | 0.79 [0.75; 0.83] | 0.59 [0.53; 0.65] |
| Mild | 0.25 [0.20; 0.30] | 0.81 [0.76; 0.86] | 0.62 [0.53; 0.71] | 0.47 [0.42; 0.52] | 1.34 [0.96; 1.88] | 0.92 [0.84; 1.01] | 1.46 [0.95; 2.23] | 0.06 [-0.04; 0.17] | 0.53 [0.50; 0.57] | 0.08 [-0.01; 0.16] |
| Moderate | 0.35 [0.30; 0.41] | 0.91 [0.87; 0.95] | 0.83 [0.76; 0.90] | 0.54 [0.48; 0.59] | 4.11 [2.63; 6.43] | 0.71 [0.64; 0.78] | 5.82 [3.46; 9.77] | 0.27 [0.17; 0.36] | 0.63 [0.60; 0.67] | 0.32 [0.23; 0.39] |
| Severe | 0.27 [0.22; 0.33] | 0.97 [0.94; 0.99] | 0.93 [0.85; 0.97] | 0.52 [0.47; 0.57] | 10.58 [4.70; 23.84] | 0.75 [0.69; 0.80] | 14.19 [6.06; 33.26] | 0.25 [0.17; 0.32] | 0.62 [0.60; 0.65] | 0.33 [0.25; 0.41] |
| **6. Visible blood in the urine** | 0.36 [0.31; 0.42] | 0.91 [0.86; 0.94] | 0.82 [0.75; 0.89] | 0.54 [0.48; 0.59] | 3.81 [2.49; 5.84] | 0.71 [0.64; 0.78] | 5.40 [3.27; 8.92] | 0.27 [0.17; 0.36] | 0.63 [0.60; 0.67] | 0.31 [0.23; 0.39] |
| Mild | 0.19 [0.14; 0.24] | 0.95 [0.92; 0.98] | 0.83 [0.71; 0.91] | 0.49 [0.44; 0.53] | 3.92 [2.10; 7.33] | 0.85 [0.80; 0.91] | 4.59 [2.34; 9.01] | 0.14 [0.06; 0.21] | 0.57 [0.54; 0.60] | 0.21 [0.12; 0.29] |
| Moderate | 0.11 [0.07; 0.15] | 0.97 [0.94; 0.99] | 0.81 [0.65; 0.92] | 0.47 [0.42; 0.51] | 3.49 [1.56; 7.80] | 0.92 [0.88; 0.97] | 3.78 [1.63; 8.78] | 0.07 [0.01; 0.13] | 0.54 [0.52; 0.56] | 0.14 [0.06; 0.23] |
| Severe | 0.07 [0.04; 0.11] | 0.98 [0.96; 1.00] | 0.83 [0.63; 0.95] | 0.46 [0.42; 0.51] | 4.07 [1.41; 11.74] | 0.95 [0.91; 0.98] | 4.30 [1.40; 12.77] | 0.05 [0.00; 0.10] | 0.53 [0.51; 0.54] | 0.13 [0.04; 0.21] |
| *Note -* 'CI' - confidence interval, 'AUC' - area under curve, 'DOR' - diagnostic odds ratio, '+LR' - positive likelihood ratio, '-LR' - negative likelihood ratio, PPV - positive predictive value, NPV - Negative predictive value, PO - positive outcome (diagnosis of AC) | | | | | | | | | | |

Supplementary Table 2. Sensitivity, specificity, positive and negative predictive values (+LR, -LR), diagnostic odds ratios (DOR), Youden’s index, area under the curve (AUC), correlation with positive outcome (PO) for diagnosis of cystitis according to the number of symptoms present. Average value [95% confidence interval]

| **1** | **Parameter** | **Sensitivity** | **Specificity** | **PPV** | **NPV** | **+LR** | **-LR** | **DOR** | **Youden's index** | **AUC** | **Correlation with PO** |
| --- | --- | --- | --- | --- | --- | --- | --- | --- | --- | --- | --- |
| 2 | At least one of three symptoms, proposed by EMA* is positive | 0.99 (0.97, 1.00) | 0.39 (0.33, 0.46) | 0.67 (0.62, 0.71) | 0.98 (0.92, 1.00) | 1.63 (1.47, 1.81) | 0.02 (0.00, 0.07) | 91.32 (22.17, 376.13) | 0.39 (0.30, 0.46) | 0.82 (0.80, 0.85) | 0.50 (0.43, 0.56) |
| 3 | At least one of four symptoms, proposed by FDA** is positive | 1.00 (0.98, 1.00) | 0.29 (0.24, 0.36) | 0.63 (0.59, 0.68) | 0.99 (0.92, 1.00) | 1.41 (1.30, 1.53) | 0.01 (0.00, 0.09) | 117.76 (16.20, 856.00) | 0.29 (0.22, 0.36) | 0.81 (0.78, 0.84) | 0.42 (0.35, 0.49) |
| 4 | At least one of six items of the "Typical" domain of the ACSS*** is positive | 1.00 (0.99, 1.00) | 0.25 (0.20, 0.32) | 0.62 (0.58, 0.67) | 0.98 (0.91, 1.00) | 1.34 (1.24, 1.45) | 0.01 (0.00, 0.10) | 97.20 (13.35, 707.86) | 0.25 (0.18, 0.32) | 0.81 (0.79, 0.83) | 0.40 (0.32, 0.47) |
| 5 | At least two of three symptoms, proposed by EMA are positive | 0.95 (0.91, 0.97) | 0.74 (0.68, 0.80) | 0.82 (0.77, 0.86) | 0.92 (0.87, 0.95) | 3.66 (2.94, 4.56) | 0.07 (0.04, 0.12) | 51.60 (28.40, 93.76) | 0.69 (0.59, 0.77) | 0.87 (0.84, 0.90) | 0.71 (0.67, 0.75) |
| 6 | At least two of four symptoms, proposed by FDA are positive | 0.98 (0.95, 0.99) | 0.63 (0.57, 0.70) | 0.77 (0.72, 0.81) | 0.96 (0.92, 0.99) | 2.67 (2.25, 3.17) | 0.03 (0.01, 0.07) | 80.42 (34.31, 188.46) | 0.61 (0.52, 0.69) | 0.86 (0.84, 0.89) | 0.67 (0.62, 0.71) |
| 7 | At least two of six items of the "Typical" domain of the ACSS are positive | 0.99 (0.97, 1.00) | 0.59 (0.52, 0.65) | 0.75 (0.70, 0.79) | 0.98 (0.94, 1.00) | 2.39 (2.05, 2.79) | 0.02 (0.01, 0.06) | 133.17 (41.45, 427.87) | 0.58 (0.49, 0.65) | 0.86 (0.84, 0.89) | 0.65 (0.59, 0.69) |
| 8 | Three of three symptoms, proposed by EMA are positive | 0.78 (0.72, 0.82) | 0.91 (0.86, 0.94) | 0.91 (0.87, 0.95) | 0.77 (0.71, 0.82) | 8.57 (5.67, 12.94) | 0.25 (0.20, 0.31) | 34.70 (20.47, 58.81) | 0.68 (0.59, 0.77) | 0.84 (0.81, 0.87) | 0.68 (0.63, 0.73) |
| 9 | At least three of four symptoms, proposed by FDA are positive | 0.91 (0.87, 0.94) | 0.83 (0.78, 0.88) | 0.87 (0.82, 0.90) | 0.88 (0.83, 0.92) | 5.39 (4.03, 7.19) | 0.11 (0.08, 0.16) | 47.29 (27.97, 79.93) | 0.74 (0.64, 0.81) | 0.87 (0.84, 0.90) | 0.74 (0.70, 0.78) |
| 10 | At least three of six items of the "Typical" domain of the ACSS are positive | 0.95 (0.92, 0.97) | 0.70 (0.64, 0.76) | 0.80 (0.75, 0.84) | 0.92 (0.87, 0.96) | 3.20 (2.62, 3.90) | 0.07 (0.04, 0.12) | 45.73 (24.93, 83.86) | 0.65 (0.56, 0.73) | 0.86 (0.83, 0.89) | 0.68 (0.64, 0.73) |
| 11 | Four of four symptoms, proposed by FDA are positive | 0.67 (0.61, 0.72) | 0.93 (0.89, 0.96) | 0.92 (0.88, 0.95) | 0.69 (0.64, 0.75) | 9.67 (5.98, 15.62) | 0.36 (0.30, 0.42) | 27.0 (15.36, 47.48) | 0.60 (0.50, 0.68) | 0.80 (0.78, 0.84) | 0.61 (0.55, 0,66) |
| 12 | At least four of six items of the "Typical" domain of the ACSS are positive | 0.88 (0.84, 0.92) | 0.85 (0.80, 0.90) | 0.88 (0.84, 0.92) | 0.85 (0.80, 0.90) | 6.01 (4.39, 8.22) | 0.14 (0.10, 0.19) | 42.99 (25.80, 71.63) | 0.73 (0.64, 0.81) | 0.87 (0.84, 0.90) | 0.73 (0.69, 0.77) |
| 13 | At least five of six items of the "Typical" domain of the ACSS are positive | 0.69 (0.63, 0.74) | 0.92 (0.88, 0.95) | 0.92 (0.87, 0.95) | 0.71 (0.65, 0.76) | 8.86 (5.65, 13.91) | 0.34 (0.28, 0.40) | 26.18 (15.22, 45.03) | 0.61 (0.51, 0.69) | 0.81 (0.78, 0.84) | 0.62 (0.56, 0.67) |
| 14 | Five of five items of the "Typical" domain of the ACSS are positive**** | 0.64 (0.58, 0.69) | 0.94 (0.90, 0.97) | 0.93 (0.88, 0.96) | 0.68 (0.62, 0.73) | 10.52 (6.29, 17.62) | 0.39 (0.33, 0.45) | 27.10 (15.00, 48.97) | 0.57 (0.48, 0.66) | 0.80 (0.77, 0.83) | 0.59 (0.53, 0.64) |
| 15 | Six of six items of the "Typical" domain of the ACSS are positive | 0.28 (0.23, 0.33) | 0.98 (0.95, 0.99) | 0.94 (0.87, 0.98) | 0.52 (0.48, 0.57) | 12.86 (5.30, 31.23) | 0.74 (0.69, 0.80) | 17.41 (6.92, 43.83) | 0.26 (0.18, 0.33) | 0.73 (0.70, 0.77) | 0.34 (0.27, 0.42) |
| 16 | At least one of three symptoms, proposed by EMA is positive + pyuria | 0.84 (0.79, 0.88) | 0.83 (0.77, 0.87) | 0.86 (0.81, 0.90) | 0.81 (0.75, 0.86) | 4.88 (3.67, 6.50) | 0.19 (0.14, 0.25) | 25.60 (16.06, 40.81) | 0.67 (0.57, 0.76) | 0.83 (0.80, 0.87) | 0.67 (0.62 0.71) |
| 17 | At least one of four symptoms, proposed by FDA is positive + pyuria | 0.85 (0.80, 0.89) | 0.79 (0.73, 0.84) | 0.83 (0.78, 0.87) | 0.81 (0.75, 0.86) | 4.00 (3.11, 5.16) | 0.20 (0.15, 0.26) | 20.46 (13.04, 32.09) | 0.63 (0.53, 0.73) | 0.82 (0.78, 0.85) | 0.64 (0.58, 0.68) |
| 18 | At least one of six items of the "Typical" domain of the ACSS is positive + pyuria | 0.85 (0.80, 0.89) | 0.76 (0.70, 0.82) | 0.81 (0.77, 0.86) | 0.80 (0.75, 0.85) | 3.58 (2.83, 4.53) | 0.20 (0.15, 0.26) | 18.11 (11.62, 28.22) | 0.61 (0.51, 0.70) | 0.81 (0.78, 0.84) | 0.62 (0.56, 0.67) |
| 19 | At least two of three symptoms, proposed by EMA are positive + pyuria | 0.80 (0.75, 0.84) | 0.91 (0.87, 0.95) | 0.92 (0.88, 0.95) | 0.79 (0.73, 0.84) | 9.28 (6.08, 14.17) | 0.22 (0.17, 0.28) | 42.40 (24.64, 72.95) | 0.71 (0.62, 0.79) | 0.85 (0.82, 0.88) | 0.71 (0.66, 0.75) |
| 20 | At least two of four symptoms, proposed by FDA are positive + pyuria | 0.83 (0.78, 0.87) | 0.88 (0.84, 0.92) | 0.90 (0.85, 0.93) | 0.81 (0.76, 0.86) | 7.15 (4.99, 10.23) | 0.19 (0.15, 0.25) | 37.49 (22.57, 62.26) | 0.71 (0.62, 0.80) | 0.85 (0.82, 0.88) | 0.71 (0.67, 0.75) |
| 21 | At least two of six items of the "Typical" domain of the ACSS are positive + pyuria | 0.84 (0.79, 0.88) | 0.86 (0.81, 0.90) | 0.88 (0.84, 0.92) | 0.82 (0.76, 0.86) | 6.11 (4.41, 8.45) | 0.18 (0.14, 0.24) | 33.33 (20.41, 54.44) | 0.70 (0.60, 0.79) | 0.85 (0.82, 0.88) | 0.70 (0.65, 0.74) |
| 22 | Three of three symptoms, proposed by EMA are positive + pyuria | 0.66 (0.61, 0.72) | 0.96 (0.93, 0.98) | 0.95 (0.92, 0.98) | 0.70 (0.65, 0.75) | 17.09 (8.96, 32.61) | 0.35 (0.30, 0.41) | 48.78 (23.98, 99.24) | 0.62 (0.53, 0.70) | 0.83 (0.80, 0.86) | 0.64 (0.58, 0.88) |
| 23 | At least three of four symptoms, proposed by FDA are positive + pyuria | 0.76 (0.71, 0.81) | 0.94 (0.91, 0.97) | 0.94 (0.91, 0.97) | 0.77 (0.71, 0.81) | 13.65 (8.02, 23.24) | 0.25 (0.20, 0.31) | 54.81 (29.40, 102.19) | 0.71 (0.62, 0.78) | 0.85 (0.83, 0.88) | 0.71 (0.66, 0.75) |
| 24 | At least three of six items of the "Typical" domain of the ACSS are positive + pyuria | 0.80 (0.75, 0.85) | 0.91 (0.86, 0.94) | 0.91 (0.87, 0.94) | 0.79 (0.74, 0.84) | 8.47 (5.67, 12.66) | 0.22 (0.17, 0.28) | 39.03 (23.03, 66.15) | 0.71 (0.61, 0.79) | 0.85 (0.82, 0.88) | 0.71 (0.66, 0.75) |
| 25 | Four of four symptoms, proposed by FDA are positive + pyuria | 0.57 (0.51, 0.63) | 0.97 (0.94, 0.99) | 0.96 (0.92, 0.99) | 0.65 (0.60, 0.70) | 22.11 (9.98, 49.03) | 0.44 (0.38, 0.50) | 50.32 (21.64, 117.04) | 0.55 (0.46, 0.62) | 0.81 (0.78, 0.84) | 0.58 (0.52, 0.63) |
| 26 | At least four of six items of the "Typical" domain of the ACSS are positive + pyuria | 0.74 (0.69, 0.79) | 0.95 (0.92, 0.98) | 0.95 (0.91, 0.98) | 0.75 (0.70, 0.80) | 15.69 (8.78, 28.04) | 0.27 (0.22, 0.33) | 58.35 (30.12, 113.04) | 0.70 (0.61, 0.77) | 0.85 (0.82, 0.88) | 0.70 (0.65, 0.74) |
| 27 | At least five of six items of the "Typical" domain of the ACSS are positive + pyuria | 0.59 (0.53, 0.64) | 0.97 (0.94, 0.99) | 0.97 (0.93, 0.99) | 0.66 (0.60, 0.71) | 22.66 (10.22, 50.21) | 0.43 (0.37, 0.49) | 53.31 (22.92, 124.01) | 0.56 (0.47, 0.63) | 0.81 (0.78, 0.84) | 0.59 (0.53, 0.64) |
| 28 | Five of six items of the "Typical" domain of the ACSS are positive + pyuria | 0.54 (0.48, 0.60) | 0.98 (0.95, 0.99) | 0.97 (0.93, 0.99) | 0.63 (0.58, 0.68) | 25.07 (10.47, 60.06) | 0.47 (0.41, 0.53) | 53.37 (21.35, 133.42) | 0.52 (0.43, 0.59) | 0.80 (0.77, 0.83) | 0.56 (0.50 0.62) |
| 29 | Six of six items of the "Typical" domain of the ACSS are positive + pyuria | 0.26 (0.21, 0.31) | 0.99 (0.96, 1.00) | 0.96 (0.89, 0.99) | 0.52 (0.47, 0.57) | 19.81 (6.33, 62.03) | 0.75 (0.70, 0.81) | 26.28 (8.16, 84.66) | 0.24 (0.17, 0.31) | 0.74 (0.71, 0.77) | 0.34 (0.26, 0.42) |
| 30 | Pyuria positive | 0.85 (0.80, 0.89) | 0.72 (0.66, 0.78) | 0.79 (0.74, 0.84) | 0.80 (0.74, 0.85) | 3.08 (2.48, 3.81) | 0.21 (0.16, 0.28) | 14.77 (9.57, 22.80) | 0.57 (0.46, 0.67) | 0.79 (0.76, 0.83) | 0.58 (0.52, 0.63) |

*Symptoms, proposed by EMA include urination frequency, urination urgency, and dysuria

**Symptoms, proposed by FDA include urination frequency, urination urgency, dysuria, and suprapubic pain

***"Typical" domain of the ACSS includes urination frequency, urination urgency, dysuria, suprapubic pain, sense of incomplete bladder emptying, and visible blood in the urine

****"Items of the Typical" domain of the ACSS, excluding visible blood in the urine

'CI' - confidence interval, 'AUC' - area under curve, 'DOR' - diagnostic odds ratio, '+LR' - positive likelihood ratio, '-LR' - negative likelihood ratio, PPV - positive predictive value, NPV - Negative predictive value, PO - positive outcome (diagnosis of AC)

Supplementary Table 3. Sensitivity, specificity, positive and negative predictive values (+LR, -LR), diagnostic odds ratios (DOR), Youden’s index, area under the curve (AUC), correlation with positive outcome (PO) for diagnosis of cystitis according to the different scores of a different number of symptoms present. Average value [95% confidence interval]

| **1** | **Parameter** | **Sensitivity** | **Specificity** | **PPV** | **NPV** | **+LR** | **-LR** | **DOR** | **Youden's index** | **AUC** | **Correlation with PO** |  |
| --- | --- | --- | --- | --- | --- | --- | --- | --- | --- | --- | --- | --- |
| **2** | Summary score of the symptoms, proposed by EMA* ≥1 | 0.99 (0.97, 1.00) | 0.39 (0.33, 0.46) | 0.67 (0.62, 0.71) | 0.98 (0.92, 1.00) | 1.63 (1.47, 1.81) | 0.02 (0.00, 0.07) | 91.32 (22.17, 376.13) | 0.39 (0.30, 0.46) | 0.82 (0.80, 0.85) | 0.50 (0.43, 0.56) |  |
| **3** | Summary score of the symptoms, proposed by FDA** ≥1 | 1.00 (0.98, 1.00) | 0.29 (0.24, 0.36) | 0.63 (0.59, 0.68) | 0.99 (0.92, 1.00) | 1.41 (1.30, 1.53) | 0.01 (0.00, 0.09) | 117.76 (16.20, 856.00) | 0.29 (0.22, 0.36) | 0.81 (0.78, 0.84) | 0.42 (0.35, 0.49) |  |
| **4** | Summary score of the "Typical" domain of the ACSS *** ≥1 | 1.00 (0.99, 1.00) | 0.25 (0.20, 0.32) | 0.62 (0.58, 0.67) | 0.98 (0.91, 1.00) | 1.34 (1.24, 1.45) | 0.01 (0.00, 0.10) | 97.20 (13.35, 707.86) | 0.25 0.18 0.32 | 0.81 (0.79, 0.83) | 0.40 (0.32, 0.47) |  |
| **5** | Summary score of five items of the "Typical" domain of the ACSS **** ≥1 | 1.00 (0.98, 1.00) | 0.27 (0.21, 0.33) | 0.63 (0.58, 0.67) | 0.98 (0.91, 1.00) | 1.36 (1.26, 1.47) | 0.01 (0.00, 0.09) | 103.94 (14.28, 756.45) | 0.26 (0.19, 0.33) | 0.81 (0.79, 0.84) | 0.41 (0.33, 0.48) |  |
| **6** | Summary score of the symptoms, proposed by EMA ≥2 | 0.97 (0.95, 0.99) | 0.69 (0.62, 0.74) | 0.79 (0.75, 0.83) | 0.95 (0.91, 0.98) | 3.09 (2.55, 3.74) | 0.04 (0.02, 0.08) | 75.42 (35.43, 160.54) | 0.66 (0.57, 0.73) | 0.87 (0.84, 0.90) | 0.70 (0.65, 0.74) |  |
| **7** | Summary score of the symptoms, proposed by FDA ≥2 | 0.99 (0.96, 1.00) | 0.58 (0.52, 0.65) | 0.74 (0.70, 0.79) | 0.97 (0.93, 0.99) | 2.36 (2.02, 2.75) | 0.02 (0.01, 0.06) | 97.77 (35.23, 271.36) | 0.57 (0.48, 0.64) | 0.86 (0.83, 0.88) | 0.64 (0.58, 0.69) |  |
| **8** | Summary score of the "Typical" domain of the ACSS ≥2 | 0.99 (0.97, 1.00) | 0.54 (0.47, 0.60) | 0.72 (0.68, 0.77) | 0.98 (0.93, 1.00) | 2.15 (1.87, 2.47) | 0.02 (0.01, 0.06) | 109.81 (34.20, 352.58) | 0.53 (0.44, 0.60) | 0.85 (0.82, 0.88) | 0.61 (0.55, 0.66) |  |
| **9** | Summary score of five items of the "Typical" domain of the ACSS ≥2 | 0.99 (0.96, 1.00) | 0.55 (0.48, 0.61) | 0.73 (0.68, 0.77) | 0.97 (0.92, 0.99) | 2.18 (1.89, 2.51) | 0.03 (0.01, 0.07) | 84.97 (30.63, 235.69) | 0.53 (0.45, 0.61) | 0.85 (0.82, 0.88) | 0.61 (0.55, 0.66) |  |
| **10** | Summary score of the symptoms, proposed by EMA ≥3 | 0.91 (0.87, 0.94) | 0.78 (0.72, 0.83) | 0.83 (0.79, 0.87) | 0.88 (0.83, 0.92) | 4.07 (3.19, 5.19) | 0.11 (0.08, 0.17) | 36.00 (21.54, 60.16) | 0.69 (0.59, 0.78) | 0.86 (0.82, 0.89) | 0.70 (0.65, 0.74) |  |
| **11** | Summary score of the symptoms, proposed by FDA ≥3 | 0.95 (0.92, 0.98) | 0.72 (0.65, 0.77) | 0.80 (0.76, 0.85) | 0.93 (0.88, 0.96) | 3.35 (2.73, 4.12) | 0.06 (0.04, 0.11) | 52.62 (28.16, 98.34) | 0.67 (0.58, 0.75) | 0.87 (0.84, 0.89) | 0.70 (0.65, 0.74) |  |
| **12** | Summary score of the "Typical" domain of the ACSS ≥3 | 0.97 (0.94, 0.99) | 0.63 (0.56, 0.69) | 0.76 (0.72, 0.81) | 0.94 (0.89, 0.97) | 2.61 (2.21, 3.09) | 0.05 (0.03, 0.10) | 52.06 (25.46, 106.46) | 0.60 (0.50, 0.68) | 0.85 (0.82, 0.88) | 0.65 (0.60, 0.70) |  |
| **13** | Summary score of five items of the "Typical" domain of the ACSS ≥3 | 0.96 (0.94, 0.98) | 0.64 (0.58, 0.70) | 0.77 (0.72, 0.81) | 0.94 (0.89, 0.97) | 2.70 (2.27, 3.21) | 0.05 (0.03, 0.10) | 49.37 (24.87, 98.00) | 0.61 (0.51, 0.69) | 0.85 (0.82, 0.88) | 0.65 (0.60, 0.70) |  |
| **14** | Summary score of the symptoms, proposed by EMA ≥4 | 0.84 (0.79, 0.88) | 0.89 (0.85, 0.93) | 0.91 (0.86, 0.94) | 0.82 (0.77, 0.87) | 7.81 (5.38, 11.36) | 0.18 (0.13, 0.23) | 44.16 (26.17, 74.56) | 0.73 (0.64, 0.81) | 0.86 (0.83, 0.89) | 0.73 (0.69, 0.77) |  |
| **15** | Summary score of the symptoms, proposed by FDA ≥4 | 0.91 (0.87, 0.94) | 0.80 (0.74, 0.85) | 0.85 (0.80, 0.88) | 0.88 (0.82, 0.92) | 4.49 (3.47, 5.81) | 0.11 (0.08, 0.17) | 39.21 (23.43, 65.61) | 0.71 (0.61, 0.79) | 0.86 (0.83, 0.89) | 0.71 (0.67, 0.75) |  |
| **16** | Summary score of the "Typical" domain of the ACSS ≥4 | 0.94 (0.91, 0.97) | 0.73 (0.67, 0.78) | 0.81 (0.76, 0.85) | 0.91 (0.86, 0.95) | 3.48 (2.81, 4.30) | 0.08 (0.05, 0.12) | 45.10 (25.22, 80.67) | 0.67 (0.58, 0.75) | 0.83 (0.86, 0.89) | 0.70 (0.65, 0.74) |  |
| **17** | Summary score of five items of the "Typical" domain of the ACSS ≥4 | 0.93 (0.90, 0.96) | 0.74 (0.68, 0.80) | 0.82 (0.77, 0.86) | 0.90 (0.85, 0.94) | 3.61 (2.90, 4.50) | 0.09 (0.06, 0.14) | 40.13 (23.14, 69.59) | 0.67 (0.58, 0.76) | 0.86 (0.83, 0.89) | 0.70 (0.65 0.74) |  |
| **18** | Summary score of the symptoms, proposed by EMA ≥5 | 0.74 (0.69, 0.79) | 0.94 (0.90, 0.97) | 0.94 (0.90, 0.97) | 0.75 (0.70, 0.80) | 12.33 (7.39, 20.58) | 0.27 (0.22, 0.33) | 45.22 (24.76, 82.59) | 0.68 (0.59, 0.76) | 0.84 (0.81, 0.87) | 0.68 (0.64, 0.73) |  |
| **19** | Summary score of the symptoms, proposed by FDA ≥5 | 0.85 (0.80, 0.89) | 0.88 (0.84, 0.92) | 0.90 (0.86, 0.93) | 0.83 (0.77, 0.87) | 7.30 (5.10, 10.44) | 0.17 (0.13, 0.23) | 42.73 (25.51, 71.58) | 0.73 (0.64, 0.81) | 0.86 (0.83, 0.89) | 0.73 (0.69, 0.77) |  |
| **20** | Summary score of the "Typical" domain of the ACSS ≥5 | 0.90 (0.86, 0.93) | 0.78 (0.73, 0.84) | 0.84 (0.79, 0.88) | 0.87 (0.81, 0.91) | 4.18 (3.26, 5.36) | 0.13 (0.09, 0.18) | 33.41 (20.26, 55.09) | 0.69 (0.59, 0.77) | 0.85 (0.82, 0.88) | 0.69 (0.65, 0.74) |  |
| **21** | Summary score of five items of the "Typical" domain of the ACSS ≥5 | 0.89 (0.85, 0.93) | 0.81 (0.75, 0.85) | 0.85 (0.80, 0.89) | 0.86 (0.81, 0.90) | 4.61 (3.54, 6.01) | 0.13 (0.09, 0.18) | 35.32 (21.45, 58.18) | 0.70 (0.60, 0.78) | 0.86 (0.83, 0.89) | 0.71 (0.66, 0.75) |  |
| **22** | Summary score of the symptoms, proposed by EMA ≥6 | 0.58 (0.52, 0.64) | 0.97 (0.93, 0.98) | 0.95 (0.91, 0.98) | 0.65 (0.60, 0.70) | 16.79 (8.44, 33.41) | 0.44 (0.38, 0.50) | 38.50 (18.31, 80.97) | 0.54 (0.45, 0.62) | 0.80 (0.77, 0.83) | 0.57 (0.51, 0.63) |  |
| **23** | Summary score of the symptoms, proposed by FDA ≥6 | 0.75 (0.70, 0.80) | 0.92 (0.88, 0.95) | 0.92 (0.88, 0.95) | 0.75 (0.70, 0.80) | 9.17 (5.93, 14.18) | 0.27 (0.22, 0.33) | 33.79 (19.68, 58.02) | 0.67 (0.57, 0.75) | 0.83 (0.80, 0.87) | 0.67 (0.62, 0.71) |  |
| **24** | Summary score of the "Typical" domain of the ACSS ≥6 | 0.87 (0.83, 0.91) | 0.88 (0.83, 0.91) | 0.90 (0.85, 0.93) | 0.85 (0.79, 0.89) | 6.96 (4.94, 9.81) | 0.15 (0.11, 0.20) | 46.92 (27.89, 78.94) | 0.75 (0.65, 0.82) | 0.87 (0.84, 090) | 0.74 (0.70, 0.78) |  |
| **25** | Summary score of five items of the "Typical" domain of the ACSS ≥6 | 0.86 (0.82, 0.90) | 0.89 (0.84, 0.93) | 0.90 (0.86, 0.94) | 0.84 (0.79, 0.88) | 7.70 (5.35, 11.10) | 0.15 (0.11, 0.21) | 49.98 (29.43, 84.88) | 0.75 (0.66, 0.83) | 0.87 (0.84, 0.90) | 0.75 (0.71, 0.78) |  |
| **26** | Summary score of the symptoms, proposed by EMA ≥7 | 0.41 (0.36, 0.47) | 0.98 (0.96, 1.00) | 0.97 (0.92, 0.99) | 0.58 (0.53, 0.63) | 24.01 (9.00, 64.07) | 0.60 (0.54, 0.66) | 40.28 (14.58, 111.27) | 0.40 (0.31, 0.47) | 0.77 (0.74, 0.80) | 0.46 (0.39, 0.53) |  |
| **27** | Summary score of the symptoms, proposed by FDA ≥7 | 0.65 (0.59, 0.71) | 0.95 (0.91, 0.97) | 0.94 (0.90, 0.97) | 0.69 (0.64, 0.74) | 12.62 (7.23, 22.03) | 0.37 (0.31, 0.43) | 34.44 (18.34, 64.68) | 0.60 (0.51, 0.68) | 0.81 (0.78, 0.84) | 0.61 (0.56, 0.67) |  |
| **28** | Summary score of the "Typical" domain of the ACSS ≥7 | 0.80 (0.75, 0.84) | 0.90 (0.85, 0.93) | 0.90 (0.86, 0.94) | 0.78 (0.73, 0.83) | 7.73 (5.27, 11.35) | 0.22 (0.18, 0.28) | 34.67 (20.76, 57.88) | 0.70 (0.60, 0.78) | 0.84 (0.81, 0.88) | 0.69 (0.64, 0.73) |  |
| **29** | Summary score of five items of the "Typical" domain of the ACSS ≥7 | 0.78 (0.73, 0.83) | 0.91 (0.86, 0.94) | 0.91 (0.87, 0.95) | 0.77 (0.72, 0.82) | 8.61 (5.70, 13.00) | 0.24 (0.19, 0.30) | 35.41 (20.87, 60.07) | 0.69 (0.59, 0.77) | 0.84 (0.81, 0.87) | 0.69 (0.64, 0.73) |  |
| **30** | Summary score of the symptoms, proposed by EMA ≥8 | 0.29 (0.24, 0.35) | 1.00 (0.98, 1.00) | 0.99 (0.94, 1.00) | 0.53 (0.49, 0.58) | 68.38 (9.59, 487.37) | 0.71 (0.66, 0.76) | 96.54 13.32 699.64 | 0.29 (0.22, 0.35) | 0.76 (0.74, 0.79) | 0.39 (0.31, 0.46) |  |
| **31** | Summary score of the symptoms, proposed by FDA ≥8 | 0.54 (0.48, 0.60) | 0.97 (0.93, 0.98) | 0.95 (0.91, 0.98) | 0.63 (0.58, 0.68) | 15.77 (7.92, 31.42) | 0.47 (0.42, 0.54) | 33.38 (15.88, 70.16) | 0.51 (0.42, 0.59) | 0.79 (0.76, 0.82) | 0.55 (0.48, 0.60) |  |
| **32** | Summary score of the "Typical" domain of the ACSS ≥8 | 0.73 (0.67, 0.78) | 0.92 (0.88, 0.95) | 0.92 (0.87, 0.95) | 0.73 (0.68, 0.78) | 8.87 (5.73, 13.73) | 0.30 (0.25, 0.36) | 29.75 (17.40, 50.88) | 0.64 (0.55, 0.73) | 0.82 (0.79, 0.86) | 0.65 (0.59, 0.69) |  |
| **33** | Summary score of five items of the "Typical" domain of the ACSS ≥8 | 0.68 (0.63, 0.74) | 0.92 (0.88, 0.95) | 0.92 (0.87, 0.95) | 0.70 (0.65, 0.75) | 8.82 (5.62, 13.84) | 0.34 (0.29, 0.41) | 25.76 (14.98, 44.29) | 0.61 (0.51, 0.69) | 0.81 (0.78, 0.84) | 0.61 (0.56, 0.66) |  |
| **34** | Summary score of the symptoms, proposed by EMA=9 | 0.14 (0.10, 0.18) | 1.00 (0.98, 1.00) | 0.97 (0.87, 1.00) | 0.49 (0.44, 0.53) | 31.88 (4.41, 230.32) | 0.87 (0.83, 0.91) | 36.78 5.01 269.88 | 0.13 0.08 0.18 | 0.74 (0.72, 0.77) | 0.26 (0.17, 0.34) |  |
| **35** | Summary score of the symptoms, proposed by FDA ≥9 | 0.41 (0.36, 0.47) | 0.98 (0.95, 0.99) | 0.96 (0.91, 0.99) | 0.58 (0.53, 0.63) | 19.21 (7.98, 46.22) | 0.60 (0.54, 0.66) | 32.08 12.82 80.25 | 0.39 (0.31, 0.47) | 0.77 (0.74, 0.80) | 0.46 (0.39, 0.52) |  |
| **36** | Summary score of the "Typical" domain of the ACSS ≥9 | 0.65 (0.59, 0.71) | 0.94 (0.91, 0.97) | 0.93 (0.89, 0.96) | 0.69 (0.63, 0.74) | 11.65 (6.82, 19.88) | 0.37 (0.31, 0.43) | 31.65 (17.19, 58.27) | 0.60 (0.50, 0.68) | 0.81 (0.78, 0.84) | 0.61 (0.55, 0.66) |  |
| **37** | Summary score of five items of the "Typical" domain of the ACSS ≥9 | 0.62 (0.56, 0.68) | 0.95 (0.92, 0.98) | 0.94 (0.90, 0.97) | 0.67 (0.62, 0.72) | 13.10 (7.31, 23.48) | 0.40 (0.34, 0.46) | 32.93 17.17 63.14 | 0.57 (0.48, 0.65) | 0.81 (0.78, 0.84) | 0.59 (0.53, 0.65) |  |
| **38** | Summary score of the symptoms, proposed by EMA ≥1+pyuria | 0.84 (0.79, 0.88) | 0.83 (0.77, 0.87) | 0.86 (0.81, 0.90) | 0.81 (0.75, 0.86) | 4.88 (3.67, 6.50) | 0.19 (0.14, 0.25) | 25.60 (16.06, 40.81) | 0.67 (0.57, 0.76) | 0.83 (0.80, 0.87) | 0.67 (0.62, 0.71) |  |
| **39** | Summary score of the symptoms, proposed by FDA ≥1+pyuria | 0.85 (0.80, 0.89) | 0.79 (0.73, 0.84) | 0.83 (0.78, 0.87) | 0.81 (0.75, 0.86) | 4.00 (3.11, 5.16) | 0.20 (0.15, 0.26) | 20.46 (13.04, 32.09) | 0.63 (0.53, 0.73) | 0.82 (0.78, 0.85) | 0.64 (0.58, 0.68) |  |
| **40** | Summary score of the "Typical" domain of the ACSS ≥1+pyuria | 0.85 (0.80, 0.89) | 0.76 (0.70, 0.82) | 0.81 (0.77, 0.86) | 0.80 (0.75, 0.85) | 3.58 (2.83, 4.53) | 0.20 (0.15, 0.26) | 18.11 (11.62, 28.22) | 0.61 (0.51, 0.70) | 0.81( 0.78, 0.84) | 0.62 (0.56, 0.67) |  |
| **41** | Summary score of five items of the "Typical" domain of the ACSS ≥1+pyuria | 0.85 (0.80, 0.89) | 0.77 (0.71, 0.82) | 0.82 (0.77, 0.86) | 0.81 (0.75, 0.86) | 3.72 (2.92, 4.73) | 0.20 (0.15, 0.26) | 19.01 (12.17, 29.70) | 0.62 (0.51, 0.71) | 0.81 (0.78, 0.85) | 0.62 (0.57, 0.67) |  |
| **42** | Summary score of the symptoms, proposed by EMA ≥2+pyuria | 0.82 (0.78, 0.87) | 0.91 (0.86, 0.94) | 0.92 (0.88, 0.95) | 0.81 (0.76, 0.85) | 9.11 (6.04, 13.75) | 0.19 (0.15, 0.25) | 47.22 (27.45, 81.24) | 0.73 (0.64, 0.81) | 0.86 (0.83, 0.89) | 0.73 (0.69, 0.77) |  |
| **43** | Summary score of the symptoms, proposed by FDA ≥2+pyuria | 0.84 (0.79, 0.88) | 0.87 (0.82, 0.91) | 0.89 (0.84, 0.92) | 0.81 (0.76, 0.86) | 6.28 (4.50, 8.74) | 0.19 (0.14, 0.24) | 33.69 (20.59, 55.13) | 0.70 (0.61, 0.79) | 0.85 (0.82, 0.88) | 0.70 (0.65, 0.74) |  |
| **44** | Summary score of the "Typical" domain of the ACSS ≥2+pyuria | 0.84 (0.79, 0.88) | 0.84 (0.78, 0.88) | 0.86 (0.82, 0.90) | 0.81 (0.76, 0.86) | 5.14 (3.83, 6.91) | 0.19 (0.14, 0.25) | 27.23 (16.99, 43.63) | 0.68 (0.58, 0.76) | 0.84 (0.81, 0.87) | 0.68 (0.63 0.72) |  |
| **45** | Summary score of five items of the "Typical" domain of the ACSS ≥2+pyuria | 0.84 (0.79, 0.88) | 0.84 (0.79, 0.89) | 0.87 (0.82, 0.91) | 0.81 (0.75, 0.86) | 5.40 (3.99, 7.33) | 0.19 (0.15, 0.25) | 28.29 (17.59, 45.50) | 0.68 (0.58, 0.77) | 0.84 (0.81, 0.87) | 0.68 (0.63, 0.73) |  |
| **46** | Summary score of the symptoms, proposed by EMA ≥3+pyuria | 0.77 (0.72, 0.82) | 0.94 (0.90, 0.96) | 0.94 (0.90, 0.96) | 0.77 (0.71, 0.81) | 11.88 (7.26, 19.47) | 0.25 (0.20, 0.31) | 48.00 (26.58, 86.70) | 0.70 (0.61, 0.78) | 0.85 (0.82, 0.88) | 0.70 (0.66, 0.74) |  |
| **47** | Summary score of the symptoms, proposed by FDA ≥3+pyuria | 0.81 (0.76, 0.85) | 0.93 (0.89, 0.96) | 0.93 (0.89, 0.96) | 0.80 (0.74, 0.84) | 11.01 (6.94, 17.47) | 0.21 (0.16, 0.26) | 52.89 (29.77, 93.97) | 0.73 (0.64, 0.81) | 0.86 (0.83, 0.89) | 0.73 (0.69, 0.77) |  |
| **48** | Summary score of the "Typical" domain of the ACSS ≥3+pyuria | 0.82 (0.77, 0.86) | 0.89 (0.85, 0.93) | 0.90 (0.86, 0.94) | 0.80 (0.75, 0.85) | 7.62 (5.24, 11.08) | 0.20 (0.16, 0.26) | 37.99 (22.73, 63.50) | 0.71 (0.62, 0.79) | 0.85 (0.82, 0.88) | 0.71 (0.66, 0.75) |  |
| **49** | Summary score of five items of the "Typical" domain of the ACSS ≥3+pyuria | 0.82 (0.77, 0.86) | 0.91 (0.86, 0.94) | 0.91 (0.87, 0.95) | 0.80 (0.75, 0.85) | 8.62 (5.77, 12.88) | 0.20 (0.16, 0.26) | 42.77 (25.12, 72.83) | 0.72 (0.63, 0.80) | 0.86 (0.83, 0.89) | 0.72 (0.67, 0.76) |  |
| **50** | Summary score of the symptoms, proposed by EMA ≥4+pyuria | 0.70 (0.64, 0.75) | 0.96 (0.93, 0.98) | 0.96 (0.92, 0.98) | 0.72 (0.67, 0.77) | 18.09 (9.49, 34.48) | 0.31 (0.26, 0.37) | 58.30 (28.58, 118.95) | 0.66 (0.57, 0.74) | 0.84 (0.81, 0.87) | 0.67 (0.62, 0.72) |  |
| **51** | Summary score of the symptoms, proposed by FDA ≥4+pyuria | 0.76 (0.71, 0.81) | 0.94 (0.90, 0.97) | 0.94 (0.90, 0.97) | 0.76 (0.71, 0.81) | 12.68 (7.60, 21.15) | 0.25 (0.20, 0.31) | 50.66 (27.64, 92.85) | 0.70 (0.61, 0.78) | 0.85 (0.82, 0.88) | 0.70 (0.66, 0.75) |  |
| **52** | Summary score of the "Typical" domain of the ACSS ≥4+pyuria | 0.80 (0.75, 0.84) | 0.92 (0.88, 0.95) | 0.93 (0.89, 0.96) | 0.79 (0.74, 0.84) | 10.31 (6.59, 16.13) | 0.22 (0.17, 0.27) | 47.56 (27.12, 83.40) | 0.72 (0.63, 0.80) | 0.86 (0.83, 0.89) | 0.72 (0.67, 0.76) |  |
| **53** | Summary score of five items of the "Typical" domain of the ACSS ≥4+pyuria | 0.79 (0.74, 0.84) | 0.94 (0.90, 0.96) | 0.78 (0.73, 0.83) | 0.90 (0.85, 0.94) | 12.21 (7.46, 19.99) | 0.23 (0.18, 0.28) | 54.25 (9.90, 98.43) | 0.72 (0.63, 0.80) | 0.86 (0.83, 0.89) | 0.72 (0.68, 0.76) |  |
| **54** | Summary score of the symptoms, proposed by EMA ≥5+pyuria | 0.62 (0.56, 0.67) | 0.97 (0.94, 0.99) | 0.97 (0.93, 0.99) | 0.67 (0.62, 0.72) | 23.88 (10.78, 52.88) | 0.39 (0.34, 0.46) | 60.82 (26.12, 141.61) | 0.59 (0.50, 0.67) | 0.82 (0.79, 0.85) | 0.62 (0.56, 0.67) |  |
| **55** | Summary score of the symptoms, proposed by FDA ≥5+pyuria | 0.71 (0.65, 0.76) | 0.97 (0.94, 0.99) | 0.97 (0.93, 0.99) | 0.73 (0.68, 0.78) | 23.49 (11.28, 48.91) | 0.30 (0.25, 0.36) | 78.23 (35.34, 173.13) | 0.68 (0.59, 0.75) | 0.85 (0.82, 0.88) | 0.69 (0.64, 0.73) |  |
| **56** | Summary score of the "Typical" domain of the ACSS ≥5+pyuria | 0.76 (0.70, 0.81) | 0.94 (0.90, 0.97) | 0.94 (0.90, 0.97) | 0.76 (0.71, 0.81) | 12.56 (7.53, 20.96) | 0.26 (0.21, 0.32) | 48.74 (26.63, 89.22) | 0.70 (0.60, 0.77) | 0.85 (0.82, 0.88) | 0.70 (0.65, 0.74) |  |
| **57** | Summary score of five items of the "Typical" domain of the ACSS ≥5+pyuria | 0.75 (0.70, 0.80) | 0.95 (0.91, 0.97) | 0.95 (0.91, 0.97) | 0.76 (0.70, 0.80) | 14.52 (8.33, 25.29) | 0.26 (0.21, 0.32) | 55.26 (29.14, 104.80) | 0.70 (0.61, 0.77) | 0.85 (0.82, 0.88) | 0.70 (0.65, 0.74) |  |
| **58** | Summary score of the symptoms, proposed by EMA ≥6+pyuria | 0.50 (0.44, 0.56) | 0.99 (0.96, 1.00) | 0.98 (0.94, 1.00) | 0.62 (0.56, 0.67) | 38.53 (12.44, 119.31) | 0.51 (0.45, 0.57) | 75.80 (23.71, 242.38) | 0.49 (0.40, 0.56) | 0.80 (0.77, 0.82) | 0.54 (0.47, 0.60) |  |
| **59** | Summary score of the symptoms, proposed by FDA ≥6+pyuria | 0.62 (0.57, 0.68) | 0.97 (0.94, 0.99) | 0.96 (0.92, 0.98) | 0.68 (0.62, 0.73) | 20.70 (9.92, 43.17) | 0.39 (0.33, 0.45) | 53.47 (24.28, 117.76) | 0.59 (0.50, 0.67) | 0.82 (0.79, 0.85) | 0.62 (0.56, 0.67) |  |
| **60** | Summary score of the "Typical" domain of the ACSS ≥6+pyuria | 0.73 (0.67, 0.78) | 0.96 (0.93, 0.98) | 0.96 (0.92, 0.98) | 0.74 (0.69, 0.79) | 18.72 (9.83, 35.67) | 0.28 (0.24, 0.34) | 65.76 (32.15, 134.48) | 0.69 (0.60, 0.76) | 0.85 (0.82, 0.88) | 0.69 (0.65, 0.74) |  |
| **61** | Summary score of five items of the "Typical" domain of the ACSS ≥6+pyuria | 0.72 (0.66, 0.77) | 0.96 (0.93, 0.98) | 0.96 (0.92, 0.98) | 0.74 (0.68, 0.78) | 18.54 (9.73, 35.33) | 0.29 (0.24, 0.35) | 63.49 (31.07, 129.75) | 0.68 (0.59, 0.75) | 0.85 (0.82, 0.88) | 0.69 (0.64, 0.73) |  |
| **62** | Summary score of the symptoms, proposed by EMA ≥7+pyuria | 0.37 (0.32, 0.43) | 0.99 (0.97, 1.00) | 0.98 (0.93, 1.00) | 0.56 (0.51, 0.61) | 43.14 (10.77, 172.89) | 0.63 (0.58, 0.69) | 68.10 16.58 279.63 | 0.36 0.28 0.43 | 0.77 (0.74, 0.80) | 0.44 (0.37, 0.51) |  |
| **63** | Summary score of the symptoms, proposed by FDA ≥7+pyuria | 0.55 (0.49, 0.61) | 0.97 (0.94, 0.99) | 0.96 (0.92, 0.99 | 0.64 (0.59, 0.69) | 21.44 (9.67, 47.54) | 0.46 (0.40, 0.52) | 46.86 (20.16, 108.95) | 0.53 (0.44, 0.60) | 0.80 (0.77, 0.83) | 0.56 (0.50, 0.62) |  |
| **64** | Summary score of the "Typical" domain of the ACSS ≥7+pyuria | 0.67 (0.61, 0.72) | 0.97 (0.93, 0.98) | 0.96 (0.92, 0.98) | 0.70 (0.65, 0.75) | 19.33 (9.74, 38.38) | 0.35 (0.29, 0.41) | 56.00 (26.53, 118.20) | 0.63 (0.54, 0.71) | 0.83 (0.80, 0.86) | 0.65 (0.59, 0.69) |  |
| **65** | Summary score of five items of the "Typical" domain of the ACSS ≥7+pyuria | 0.65 (0.59, 0.70) | 0.97 (0.94, 0.99) | 0.96 (0.93, 0.99) | 0.69 (0.64, 0.74) | 21.40 (10.26, 44.61) | 0.37 (0.31, 0.43) | 58.56 (26.56, 129.08) | 0.62 (0.53, 0.69) | 0.83 (0.80, 0.86) | 0.63 (0.58, 0.68) |  |
| **66** | Summary score of the symptoms, proposed by EMA ≥8+pyuria | 0.27 (0.22, 0.32) | 1.00 (0.98, 1.00) | 0.99 (0.93, 1.00) | 0.53 (0.48, 0.57) | 62.13 (8.71, 443.41) | 0.74 (0.69, 0.79) | 84.36 (11.63, 612.03) | 0.26 (0.19, 0.32) | 0.76 (0.74, 0.79) | 0.37 (0.30, 0.45) |  |
| **67** | Summary score of the symptoms, proposed by FDA ≥8+pyuria | 0.47 (0.41, 0.53) | 0.99 (0.96, 1.00) | 0.98 (0.94, 1.00) | 0.60 (0.55, 0.65) | 36.63 (11.82, 113.50) | 0.53 (0.48, 0.60) | 68.70 (21.48, 219.69) | 0.46 (0.38, 0.53) | 0.79 (0.76, 0.82) | 0.52 (0.45, 0.58) |  |
| **68** | Summary score of the "Typical" domain of the ACSS ≥8+pyuria | 0.62 (0.56, 0.67) | 0.97 (0.94, 0.99) | 0.96 (0.92, 0.98) | 0.67 (0.62, 0.72) | 20.47 (9.81, 42.69) | 0.39 (0.34, 0.46) | 51.90 (23.57, 114.27) | 0.59 (0.50, 0.66) | 0.82 (0.79, 0.85) | 0.61 (0.55, 0.66) |  |
| **69** | Summary score of five items of the "Typical" domain of the ACSS ≥8+pyuria | 0.58 (0.52, 0.63) | 0.97 (0.94, 0.99) | 0.96 (0.92, 0.98) | 0.65 (0.60, 0.70) | 19.07 (9.13, 39.83) | 0.44 (0.38, 0.50) | 43.57 (19.81, 95.81) | 0.55 (0.45, 0.62) | 0.80 (0.78, 0.83) | 0.58 (0.52, 0.63) |  |
| **70** | Summary score of the symptoms, proposed by EMA ≥9+pyuria | 0.12 (0.09, 0.17) | 1.00 (0.98, 1.00) | 0.97 (0.85, 1.00) | 0.48 (0.44, 0.53) | 28.61 (3.95, 207.29) | 0.88 (0.84, 0.92) | 32.48 (4.41, 238.99) | 0.12 (0.06, 0.17) | 0.74 (0.72, 0.76) | 0.24 (0.16, 0.32) |  |
| **71** | Summary score of the symptoms, proposed by FDA ≥9+pyuria | 0.36 (0.31, 0.42) | 1.00 (0.98, 1.00) | 0.99 (0.95, 1.00) | 0.56 (0.51, 0.61) | 83.85 (11.79, 596.33) | 0.64 (0.59, 0.70) | 130.73 (18.07, 945.91) | 0.36 (0.28, 0.42) | 0.77 (0.75, 0.80) | 0.44 (0.37, 0.51) |  |
| **72** | Summary score of the "Typical" domain of the ACSS ≥9+pyuria | 0.56 (0.50, 0.62) | 0.97 (0.94, 0.99) | 0.96 (0.92, 0.99) | 0.64 (0.59, 0.69) | 21.57 (9.73, 47.84) | 0.45 (0.40, 0.52) | 47.53 (20.44, 110.51) | 0.53 (0.44, 0.61) | 0.80 (0.77, 0.83) | 0.57 (0.51, 0.62) |  |
| **73** | Summary score of five items of the "Typical" domain of the ACSS ≥9+pyuria | 0.53 (0.47, 0.59) | 0.98 (0.95, 0.99) | 0.97 (0.93, 0.99) | 0.63 (0.57, 0.68) | 24.42 (10.19, 58.52) | 0.48 (0.43, 0.55) | 50.44 (20.18, 126.09) | 0.50 (0.42, 0.58) | 0.80 (0.77, 0.83) | 0.55 (0.48, 0.61) |  |
|  | | | | | | | | | | | | |

*Symptoms, proposed by EMA include urination frequency, urination urgency, and dysuria

**Symptoms, proposed by FDA include urination frequency, urination urgency, dysuria, and suprapubic pain

***Typical" domain of the ACSS includes urination frequency, urination urgency, dysuria, suprapubic pain, sense of incomplete bladder emptying, and visible blood in the urine Items of the

**Items of the Typical" domain of the ACSS, excluding visible blood in the urine

'CI' - confidence interval, 'AUC' - area under curve, 'DOR' - diagnostic odds ratio, '+LR' - positive likelihood ratio, '-LR' - negative likelihood ratio, PPV - positive predictive value, NPV - Negative predictive value, PO - positive outcome (diagnosis of AC)
